# Beware batch culture: Seasonality and niche construction predicted to favor bacterial adaptive diversification

**DOI:** 10.1371/journal.pcbi.1005459

**Published:** 2017-03-30

**Authors:** Charles Rocabert, Carole Knibbe, Jessika Consuegra, Dominique Schneider, Guillaume Beslon

**Affiliations:** 1 Univ. de Lyon, CNRS, INRIA, INSA-Lyon, UCB Lyon 1, LIRIS UMR5205, Lyon, France; 2 Univ. Grenoble Alpes, Laboratoire Techniques de l’Ingénierie Médicale et de la Complexité - Informatique, Mathématiques et Applications, Grenoble (TIMC-IMAG), Grenoble, France; 3 Centre National de la Recherche Scientifique (CNRS), TIMC-IMAG, Grenoble, France; University of New South Wales, AUSTRALIA

## Abstract

Metabolic cross-feeding interactions between microbial strains are common in nature, and emerge during evolution experiments in the laboratory, even in homogeneous environments providing a single carbon source. In sympatry, when the environment is well-mixed, the reasons why emerging cross-feeding interactions may sometimes become stable and lead to monophyletic genotypic clusters occupying specific niches, named ecotypes, remain unclear. As an alternative to evolution experiments in the laboratory, we developed Evo^2^Sim, a multi-scale model of *in silico* experimental evolution, equipped with the whole tool case of experimental setups, competition assays, phylogenetic analysis, and, most importantly, allowing for evolvable ecological interactions. Digital organisms with an evolvable genome structure encoding an evolvable metabolic network evolved for tens of thousands of generations in environments mimicking the dynamics of real controlled environments, including chemostat or batch culture providing a single limiting resource. We show here that the evolution of stable cross-feeding interactions requires seasonal batch conditions. In this case, adaptive diversification events result in two stably co-existing ecotypes, with one feeding on the primary resource and the other on by-products. We show that the regularity of serial transfers is essential for the maintenance of the polymorphism, as it allows for at least two stable seasons and thus two temporal niches. A first season is externally generated by the transfer into fresh medium, while a second one is internally generated by niche construction as the provided nutrient is replaced by secreted by-products derived from bacterial growth. In chemostat conditions, even if cross-feeding interactions emerge, they are not stable on the long-term because fitter mutants eventually invade the whole population. We also show that the long-term evolution of the two stable ecotypes leads to character displacement, at the level of the metabolic network but also of the genome structure. This difference of genome structure between both ecotypes impacts the stability of the cross-feeding interaction, when the population is propagated in chemostat conditions. This study shows the crucial role played by seasonality in temporal niche partitioning and in promoting cross-feeding subgroups into stable ecotypes, a premise to sympatric speciation.

## Author summary

Stable bacterial cross-feeding interactions, where one strain feeds on the waste of the other, are important to understand, as they can be a first step toward bacterial speciation. Their emergence is commonly observed in laboratory experiments using *Escherichia coli* as a model organism. Yet it is not clear how cross-feeding interactions can resist the invasion of a fitter mutant when the environment contains a single resource since there seems to be a single ecological niche. Here, we used another kind of “model organism”, a digital one, allowing for detailed and fast investigations, and providing a way to disentangle generic evolutionary mechanisms from specificities associated with *E. coli*. Digital organisms with evolvable genomes and metabolic networks compete for resources in conditions mimicking laboratory evolution experiments. In chemostat simulations, although cross-feeding interactions regularly emerged, selective sweeps regularly purged the population of its diversity. By contrast, batch culture allowed for much more stable cross-feeding interactions, because it includes seasons and thus distinct temporal niches, thereby favoring the adaptive diversification of proto-species.

## Introduction

Stable metabolic cross-feeding interactions between microbial strains are commonly observed in nature [1–4]. For example, nitrification, an important step of the nitrogen cycle, is carried out in consecutive steps by several bacterial species maintaining cross-feeding interactions [3]. In laboratory experiments, microbial populations also demonstrated their ability to quickly establish metabolic cross-feeding interactions between morphotypes [5–13].

An important question, at the crossroads between ecology and evolution, is the evolutionary stability of such cross-feeding polymorphisms, because they are often considered to be the first steps toward speciation. According to Cohan [14], the species concept in bacteria should not rely on the named species of systematics but on the notion of *ecotype*, which itself relies on the ecological and evolutionary dynamics of the subpopulations. Two bacterial subpopulations may be considered as different ecotypes if they form monophyletic clusters, occupy different ecological niches and if periodic selection purges diversity in one subpopulation independently from the other [14]. A cross-feeding polymorphism therefore leads to adaptive diversification and ultimately to speciation when it is stable enough to resist the invasion of a mutant that would otherwise take over the whole population.

If the environment is spatially structured, the stabilization of new ecotypes that emerged after an adaptive diversification event is facilitated by the locality of environmental conditions and frequency-dependent interactions. This mechanism of allopatric (or micro-allopatric) divergence is well-known, since ecotypes can escape competitive exclusion in their local niches [14]. For example, *Pseudomonas fluorescens* populations have been shown to produce adaptive diversification events in spatially heterogeneous environments, but not in homogenized conditions [5, 6].

Microbial populations can also exhibit adaptive diversification in sympatry, when the environment is homogeneous with a single carbon source. In this case, the stability of ecotypes is maintained by frequency-dependent interactions, often due to cross-feeding interactions, as observed in the Long-Term Evolution Experiment with *Escherichia coli* (LTEE [15]). In this ongoing experiment, 12 populations are being independently propagated in a constant glucose-limited environment in batch culture since 1988. The experiment reached 66,000 generations at the time of this writing. Every day, 1% of the population is transferred into fresh medium such that each population experiences a daily cycle of feast and famine phases. In one of the 12 populations, a long-term polymorphism has been observed [11]. Two ecotypes, named S and L (for Small and Large, related to their respective colony sizes on plate), evolved from a common ancestor before generation 6,500. The L ecotype grows efficiently on glucose, while the S ecotype mainly grows on acetate, a by-product secreted by L [16]. Experiments showed that the interaction between S and L ecotypes relies on negative frequency-dependent selection, each ecotype having a selective advantage when rare. This balanced polymorphism is now stable for more than 55,000 generations [11]. It was also shown that S and L ecotypes specialized in their own niches, the L ecotype increasing its ability to grow on glucose but not on acetate, and conversely for the S ecotype [16].

The evolutionary stability of this polymorphism may be explained by the temporal niche partitioning that arises from the periodic transfers into fresh medium [17]. A first season starts immediately after a transfer, when the environment contains mostly glucose. The L ecotype grows during this season, consumes glucose and secretes acetate, thereby generating a second season where the environment contains mostly acetate and supports the growth of the S ecotype.

Yet several experiments have shown that microbial populations can also evolve cross-feeding interactions in a chemostat in a few tens of generations [7, 8, 10]. Those interactions appear to be stable over a few hundreds of generations [7, 8, 10]. In chemostat, there is no obvious spatial or temporal niche partitioning and it is thus intriguing that the dynamics predicted by the competitive exclusion principle has not been observed so far. Indeed, one would expect a mutant to eventually appear, which would either completely degrade glucose or feed on both glucose and acetate, thereby outcompeting the specialized ecotypes. It has been proposed that energy constraints and flux optimization principles prevent competitive exclusion, thereby stabilizing the polymorphism [18, 19]. However, experimental evolution in chemostat has generally been performed for only a few hundreds of generations (up to 1,900 generations in [7]), precluding the possibility to confirm this statement on a longer term.

Thus, as a step to better understand how cross-feeding, niche construction and seasonality contribute to microbial diversification, we addressed here the following question: What makes emerging cross-feeding interactions stable in the long-term, in single carbon source batch culture or chemostat experiments?

While experimental evolution provides a very precise picture of evolution, it remains a long and costly process. An alternative approach consists in simulating evolution in a computer. *In Silico* Experimental Evolution (ISEE), where digital organisms are evolved for tens of thousands of generations, reproduces the environmental conditions of experimental evolution [20]. Like in the wet approach, it is possible to simulate several independent populations to understand the respective importance of general laws and historical contingencies. In addition, ISEE provides an exhaustive fossil record and, more importantly, allows for “impossible experiments” [21], like saving the fitness at full resolution for tens of thousands of generations, or changing any parameter (mutation rates, environment fluxes) at will.

We developed Evo^2^Sim, a multi-scale computational model of *in silico* experimental evolution. Evo^2^Sim allows us to address many questions raised by experimental evolution [20]. Typically, we can use it to investigate how evolution shapes the different organization levels of an organism (*e.g.*, genome size, complexity of the regulation network and metabolic network) and of an ecosystem (polymorphism, speciation) depending on global parameters such as environmental conditions or mutation rates. Here, we tested which environmental conditions can lead to stable adaptive diversification events, by reproducing the resource dynamics of experimental evolution setups like chemostat and batch culture.

Previous mathematical works have already studied the conditions of interspecific coexistence via resource partitioning [22], and of cross-feeding interactions [18, 23, 24], during one or more competition episodes. Stewart and Levin [22] studied the conditions of coexistence of several ecotypes in batch culture and chemostat. However, they focused on a single episode of competition between preexisting strains without modeling a random mutational process. Moreover, the strains were not allowed to cross-feed on by-products of other strains. Rozen et al. [13] and Ribeck and Lenski [25] modeled analytically the cross-feeding interaction between S and L ecotypes in the LTEE, showing the existence of negative frequency-dependence in batch conditions. These models also did not include a mutational process. Gudelj and colleagues [24] studied the short-term dynamics of two competitors in various environmental conditions including batch and chemostat, and showed that stable cross-feeding was possible, depending on initial competitors frequency and resource abundance. Again, this model did not include the mutational process. Other mathematical studies introduced a simplified evolutionary dynamics, by computing successions of competition episodes and introduction of fit mutants. For example, Pfeiffer and Bonhoeffer [18] studied the conditions of emergence of stable cross-feeding in chemostat conditions, when a trade-off on ATP production is introduced on abstract metabolic pathways. Doebeli [23] compared the conditions of emergence of cross-feeding polymorphism in chemostat and batch culture. The authors concluded that the evolution of cross-feeding is more likely in chemostat than in batch culture. However, this model forced a trade-off between consumption rates of glucose and acetate, forbidding the emergence of a generalist mutant. Two rates are evolvable but only the glucose consumption rate is mutable, as the acetate rate is deduced from the glucose rate. The rate at which acetate is secreted is constant (*i.e.*, it does not depend on glucose consumption, which could affect the generality of the conclusions). Thus, none of the previous models take into account a realistic random mutational process, and none of them explicitly models the genomic level. Indeed, it is difficult to include a competition process as well as realistic mutational dynamics in a single mathematical model. Another approach consists in simulating evolution with individual-based models.

Computational models of *in silico* experimental evolution have already been used to explore the evolution of cross-feeding interactions. Johnson and Wilke [26] used the Avida software [27] to study the evolution of resource competition between two digital species coexisting via mutualistic cross-feeding in a closed environment, with only two possible metabolites. However, they did not test the influence of the environmental dynamics. Williams and Lenton [28] used an individual-based evolutionary model to explore the stability of connected ecosystems undergoing cross-feeding and “evolutionary regime shifts”. Yet, the genotype-to-phenotype mapping of their organisms was rather simple (fixed size arrays defining the affinity of the organism for each resource), thus not allowing to study the effects of ecological dynamics on genome and metabolic network structures. Crombach and Hogeweg [29] and Boyle et al. [30] studied the evolution of resource cycling and its stability. In the first model [29], the resource cycling was imposed by the system. In the second model [30], the environment was strongly structured (patches of individuals with random migration events), such that it was not possible to study sympatric diversification. Chow and colleagues [31] used Avida [27] to explore the relation between productivity and diversity in a digital ecosystem under mixed influx of nine pre-defined resources, while Gerlee and Lundh [19] explained the maintenance of cross-feeding interactions in a microbial population by energy and efficiency constraints on metabolic fluxes. To do so, they developed an individual-based model evolving simple binary strings, thereby precluding evolvable interactions between the different organization levels of an organism, and their possible effects on the ecological dynamics. Gerlee and Lundh [32] also related ecosystem productivity to energy-uptake efficiency, with the same type of individual-based model as in [19]. Recently, Liu and Sumpter [33] used an individual-based model evolving artificial ecosystems relying on a “number soup”: In this model, each species perform one modular addition transforming specific numbers into others, immediately available for other species. With their model, authors showed that artificial ecosystems always self-organize to consume all the available resources. While stable cross-feeding, and reciprocal cross-feeding, are common evolutionary outcomes in their model, authors also show that whole population extinctions sometimes occur, even without external perturbations. Yet, the absence of complex and evolvable genotype-to-phenotype map in their model precludes the possibility to get insights into the influence of ecosystem evolution on the structure of the organisms. Finally, Großkopf et al. [16] predicted the adaptive diversification event leading to S and L ecotypes in the LTEE, by mixing flux balance analysis (FBA) and *in silico* evolution in a single model. By modeling the evolution of reaction rates in the metabolic network of *Escherichia coli*, they demonstrated that the emergence of a stable cross-feeding similar to S and L interaction is highly probable in the LTEE conditions. However, in their model, digital organisms are highly constrained (there is no innovation, *e.g.* new by-products cannot appear in the evolutionary process). To the best of our knowledge, none of these individual-based models compared the evolution of stable cross-feeding in different experimental setups, such as batch culture or chemostat.

To sum up, we were not able to find in the literature models that combine: **(i)** an explicit mutational process along with the modeling of natural selection and drift, **(ii)** evolvability at all organization levels (genome structure, metabolic network, number of reactions, number of metabolites, reaction rates, …), and **(iii)** a comparison between batch culture and chemostat.

Our results show that *stable* cross-feeding interactions are favored in batch culture, owing to the seasonality of the environment. In continuous culture, the absence of seasonality precludes niche construction and leads to competitive exclusion, even if the population is initially composed of two ecotypes maintaining frequency-dependent interactions. We also demonstrate that the long-term evolution of a stable cross-feeding interaction in batch culture leads to character displacement [16, 34], at the level of the metabolic network but also of the genome structure. This difference of genome structure between the two ecotypes has an impact on the further stability of the cross-feeding interaction when the population is propagated into continuous culture.

### Model

**Fig 1:**
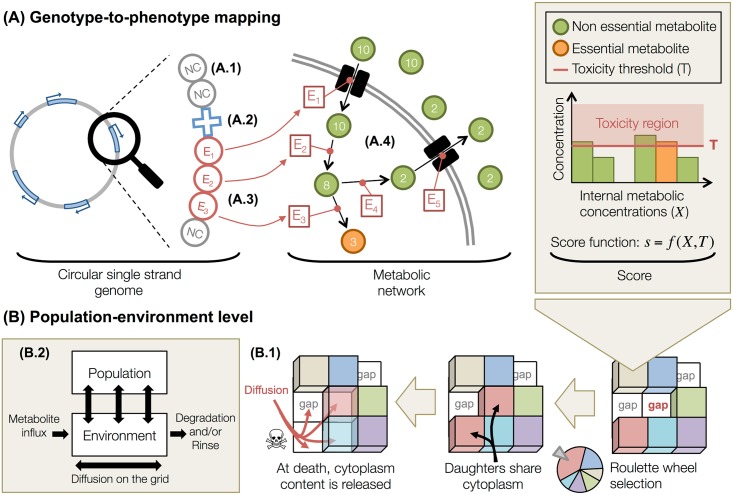
Presentation of the model. The genotype-to-phenotype mapping, as well as the population and environment, are schematized here. **(A)** Description of the genotype-to-phenotype mapping. Organisms own a coarse-grained genome that contains genomic units. **(A.1)** Non-coding units (NC, grey circles) are not functional. The arrangement of the genomic units on the circular single strand defines functional regions, where a promoter (P, blue cross, **A.2**) controls the expression of all contiguous enzyme units (E, red circles), thereby allowing for operons. **(A.3)** When enzyme units are expressed, they contribute to the metabolic network. **(A.4)** Enzymes perform metabolic reactions in the cytoplasm, or pump metabolites in or out (see the description of the metabolic network below). The score of an organism is computed from its “essential metabolites” (see the description of the score function below). Lethal toxicity thresholds are applied to each metabolic concentration and preclude organisms to accumulate resources. **(B)** Description of the population and environment levels. Organisms are placed on a 2D toroidal grid, and compete for resources and space. **(B.1)** When an organism dies, it leaves its grid spot empty and organisms in the Moore neighborhood (if any) compete to divide in the available spot. The competition is based on scores, a minimal threshold being applied on scores to preclude worst organisms to divide. At division, daughters share cytoplasm content (enzymes and metabolites). At death, metabolites from the cytoplasm are released in the local environment and diffuse on the grid. **(B.2)** At the largest scale, the population evolves in the environment by uptaking, transforming and releasing metabolites. Metabolites then diffuse and are optionally degraded. This interaction between the population and its environment allows for the evolution of complex ecological situations.

Evo^2^Sim is a multi-scale and individual-based computational model. Digital bacterial-like organisms own a coarse-grained genome that contains genomic units encoding a simplified metabolic network. The organisms evolve on a two-dimensional toroidal grid (the environment), uptaking, transforming and releasing metabolites, and dividing in the presence of empty spots or dying. Extracellular metabolites diffuse across the grid spots. In this model, metabolites are implicit molecules identified by a tag $\in N^{*}$. The model is described in more details below, and summarized in Fig 1. The source code is written in C++. All the material necessary to replay experiments (software, parameter files, strain backups, …) is freely available at http://www.evoevo.eu/adaptive-diversification-simulations/. The latest version of Evo^2^Sim is available at http://www.evoevo.eu/evo2sim-software/.

#### Genome structure

The genome is a circular single-stranded sequence of genomic units, inspired from [35, 36]. Genomic units belong to three different types: non-coding units (NC), promoter units (P), and enzyme coding units (E). The order of the units in the genome determines the existence of functional regions, meaning that not all sequences of units are functional. The functional regions of a genome are those that have the following pattern: a promoter (P) followed by one or more enzyme coding units (E). A promoter can thus control several coding units, as bacterial operons. The first genomic unit that is not enzyme coding interrupts transcription and marks the end of the functional region.

Non-coding units (NC) have no particular function. They constitute the non-coding part of the genome. Promoter units (P) contain a floating-point number *β* ∈ [0.0, 1.0] representing the production rate of the protein(s) depending on the promoter. All the parameters and their units are listed in S1 Table. Enzyme coding units (E) contain two integers *s* and $p\in N^{*}$, indicating the tag of the substrate and product respectively, two floating-point numbers *k*_*cat*_ ∈ ±[10^−3^, 10^−1^], and the ratio *k*_*cat*_/*K*_*M*_ ∈ [10^−5^, 10^−3^] describing the enzymatic kinetics (see the description of the metabolic network below). In the special case where *s* = *p*, the enzyme is considered as a pump, actively pumping in (or out) the metabolite *s* if *k*_*cat*_ is positive (or negative, respectively). Initial genomes of 50 genomic units are generated. These genomes contain ten P and ten E, all with random positions and attribute values.

Upon cell division, the parental genome is replicated with mutations in the two daughter cells. Each genomic unit can undergo point mutations, meaning here changes in the numbers it contains, like the values of *s*, *p*, *k*_*cat*_ and *k*_*cat*_/*K*_*M*_ for an E. Each unit attribute mutates at a rate of 10^−3^ per attribute per replication. For the substrate/product tags, a mutation consists in randomly incrementing/decrementing *s* or *p* respectively. For *k*_*cat*_ or *k*_*cat*_/*K*_*M*_, a random number drawn from N(0,0.1) is added to the decimal logarithm of the parameter. *β* mutates by adding a random number drawn from N(0,0.1). A genomic unit can also undergo a type transition from any unit type to any other at a predefined rate, set here to 10^−3^ per genomic unit per replication. All types of genomic units are actually implemented as a tuple containing all possible attributes, like (unit_type, *β*, *s*, *p*, *k*_*cat*_, *k*_*cat*_/*K*_*M*_). The unit type tells us which parameters are functionally relevant and the others are free to mutate neutrally.

The genome can also undergo rearrangements affecting segments of any number of genomic units. There are four types of rearrangements: duplications, deletions, translocations and inversions. All rearrangement rates are set to 10^−3^ per genomic unit per replication, hence the number of rearrangements is related to the genome size thereby limiting genome expansion [37]. The breakpoints for each rearrangement are randomly drawn in the whole genome. In real genomes, spontaneous rearrangement breakpoints have no reason to lie exactly between two of our genomic units and could thus break our genomic units. To model that with our coarse-grained genome representation, we alter the content of the two genomic units that are adjacent to a rearrangement breakpoint. Suppose for example that a deletion joins two genomic units, one containing the attributes (unit_type_1_, *β*_1_, *s*_1_, *p*_1_, *k*_*cat*1_, (*k*_*cat*_/*K*_*M*_)_1_) and the other the attributes (unit_type_2_, *β*_2_, *s*_2_, *p*_2_, *k*_*cat*2_, (*k*_*cat*_/*K*_*M*_)_2_). Then for each attribute, there is a probability of 10^−3^ for the value in unit 1 to be exchanged with the value in unit 2. Both units could for example exchange their values of *s*, thereby leading to (unit_type_1_, *β*_1_, *s*_2_, *p*_1_, *k*_*cat*1_, (*k*_*cat*_/*K*_*M*_)_1_) and (unit_type_2_, *β*_2_, *s*_1_, *p*_2_, *k*_*cat*2_, (*k*_*cat*_/*K*_*M*_)_2_).

#### Metabolic network

Gene products can either be pumps, pumping metabolites from or to the growth medium, or enzymes performing catalytic transformations in the metabolic space.

Let us consider an enzyme in the cytoplasm, that catalyzes one specific reaction *s* → *p*, with $s\in N^{*}$ and $p\in N^{*}$ being the substrate and the product of a Michaelis-Menten-like reaction, respectively. The variation in concentrations [*E*], [*s*] and [*p*] over time are then driven by Eq 1:

$$\begin{array}{c} \left\{\begin{array}{c} \frac{d[E]}{dt}=\beta -\phi \left[E\right]\\ \frac{d[s]}{dt}=-\frac{k_{cat}\left[E\right]\left[s\right]}{K_{M}+\left[s\right]}\\ \frac{d[p]}{dt}=\frac{k_{cat}\left[E\right]\left[s\right]}{K_{M}+\left[s\right]} \end{array}\right. \end{array}$$ (1)

where *β* is the basal production rate specified in the promoter unit, *ϕ* is the enzyme degradation rate (set to 0.1 per centi-time-step for all enzymes here, with 1 centi-time-step = 0.01 time-steps), *K*_*M*_ and *k*_*cat*_ are the kinetic attributes of the enzyme (*K*_*M*_ being deduced from *k*_*cat*_ and *k*_*cat*_/*K*_*M*_ attributes).

Pumps are treated here as special enzymes for which [*s*] and [*p*] describe the internal and external concentrations of the same metabolite. If *k*_*cat*_ is positive (resp. negative), [*s*] is the external (resp. internal) concentration of the metabolite and [*p*] the internal (resp. external) concentration. The dynamics of metabolic concentrations [*s*] and [*p*] are thus also driven by Eq 1 when the gene product is a pump.

Each organism has an ODE (Ordinary Differential Equation) system that keeps track of: **(i)** the concentrations of all metabolites inside the organism, *i.e.*, internal concentrations, **(ii)** the concentrations of all metabolites at the organism’s location on the grid, *i.e.*, external concentrations, and **(iii)** the concentrations of all proteins (pumps and enzymes) in the cytoplasm. For a very simple organism whose genome merely encodes one pump importing metabolite #10 into the cell, and one enzyme converting #10 to #7, the ODE system would read:

$$\begin{array}{c} \left\{\begin{array}{c} \frac{d[\text{Pump}]}{dt}=\beta^{\text{Pump}}-\phi \left[\text{Pump}\right]\\ \frac{d[\text{Enzyme}]}{dt}=\beta^{\text{Enzyme}}-\phi \left[\text{Enzyme}\right]\\ \frac{d[\#10_{\text{external}}]}{dt}=-\frac{k_{cat}^{\text{Pump}}\left[\text{Pump}\right]\left[\#10_{\text{external}}\right]}{K_{M}^{\text{Pump}}+\left[\#10_{\text{external}}\right]}\\ \frac{d[\#10_{\text{internal}}]}{dt}=\frac{k_{cat}^{\text{Pump}}\left[\text{Pump}\right]\left[\#10_{\text{external}}\right]}{K_{M}^{\text{Pump}}+\left[\#10_{\text{external}}\right]}-\frac{k_{cat}^{\text{Enzyme}}\left[\text{Enzyme}\right]\left[\#10_{\text{internal}}\right]}{K_{M}^{\text{Enzyme}}+\left[\#10_{\text{internal}}\right]}\\ \frac{d[\#7_{\text{internal}}]}{dt}=\frac{k_{cat}^{\text{Enzyme}}\left[\text{Enzyme}\right]\left[\#10_{\text{internal}}\right]}{K_{M}^{\text{Enzyme}}+\left[\#10_{\text{internal}}\right]} \end{array}\right. \end{array}$$ (2)

The number of equations in the ODE system generally differs across individuals within a population because it depends on the number of functional genes, and chromosomal rearrangements like duplications and deletions can alter gene number. In practice, the size of the ODE system goes from tens to thousands of equations depending on the individual. Similarly, the parameter values of the ODE system also vary across individuals, as they are encoded in the organism’s genes and thus result from the mutation process.

Initially, in the individuals used to seed a run at time-step 0, each protein starts at its equilibrium concentration *β*/*ϕ*, and each metabolite starts with an internal concentration of 0.0 ACU (Arbitrary Concentration Unit). At time-step 0, for all grid spots, external concentrations are initialized to 0.0 ACU for all nutrients except for metabolite #10 (the exogenous carbon source). Between time-steps 0 and 1, the ODE system computes the dynamics of the metabolite and protein concentrations, using the adaptive Runge-Kutta-Cash-Karp method (RKCK), during 100 centi-time-steps. In organisms that possess a pump for metabolite #10, this metabolite will enter the cell. If the genome of this organism also encodes an enzyme to transform #10 into #7 for example, then the internal concentrations will show an accumulation of #7. At time-step 1, each organism will either die, divide or just survive (see paragraph “Population and environment” below for details). If the organism merely survives without dividing, its current internal concentrations are used as initial conditions for the computation of the next 100 centi-time-steps (*i.e.*, for the transition from time-step 1 to 2). If the organism divides, each of the two daughter cells inherits half of each metabolite and each protein amounts. These will constitute the initial conditions for each cell’s ODE system for the next 100 centi-time-steps. If the organism dies, its internal content is released into the environment, thereby increasing the local external concentrations. As the metabolites can diffuse across the grid, the metabolites produced by the dead cell, like metabolite #7, will become available to the neighboring cells, which will thus be able to feed on both #10 and #7, if they own the corresponding pumps. This process is repeated for each transition from time-step *t* to time-step *t* + 1.

Thus, when *e.g.*, a 32 × 32 grid is full of organisms (see the description of the experimental protocol below), a time-step involves the computation of about a thousand different ODE systems, each of them containing from tens to thousands of equations depending on gene number.

#### Score function

Some metabolites are essential for an organism’s replication. Here, we arbitrarily define as essential the metabolites whose tag is a prime number. The score of an organism is then simply defined as the sum of its internal concentrations of essential metabolites. However, to prevent organisms from producing a single specific prime number in huge quantities, we also define lethal toxicity thresholds for both essential and non essential metabolites. Here these toxicity thresholds are set to 1.0 ACU for all metabolites.

#### Population and environment

Organisms evolve on a two-dimensional toroidal grid, each spot containing at most one organism. The physical environment is described at the grid level: each grid spot contains external metabolites, each with its concentration. These external metabolites diffuse with a diffusion parameter *D* = 0.1 gridstep^2^.time-step^-1^, meaning that a fraction *D* of each metabolite present at one location will diffuse to each of the eight neighboring grid spots at each time-step. The discrete diffusion equation we are using is inspired from [38]. External metabolites are also degraded with a degradation rate *D*_*g*_, meaning that a fraction *D*_*g*_ of each metabolite at each location will disappear at each time-step. We make the simplifying assumption that there are no enzymatic reaction in the environment, and thus that metabolite transformation only occurs inside the organisms. Organisms compete for the external metabolites to produce offspring in empty spots. They interact with their local environment by pumping metabolites in and out and releasing their metabolic content at death. At each time-step, organisms are evaluated and either killed, updated or replicated depending on their current state:

1. If the organism does not die and cannot divide (*e.g.*, because there is no free space in its neighborhood), its metabolic network is updated, and its score is computed. If lethal toxicity thresholds are reached, the organism dies (see point 2);
2. Organisms can also die randomly with a probability following a Poisson law of parameter *p*_*death*_ = 0.02 per organism per time-step. At death, the metabolic content is released into the local environment;
3. For each empty grid spot, all living organisms in the Moore neighborhood whose score is higher than a minimum score of 10^−3^ ACU compete. The organism having the best score in the neighborhood is allowed to divide if it did not replicate previously at the same time-step (such that any dividing cell generates at most two daughters per time-step).

### Experimental protocol

In all our simulations, the environment provided one primary resource with tag *m*_*exo*_ = #10. To initialize an evolutionary run, the entire grid was populated with individuals having random genomes (different for each individual). This initial population was allowed to evolve for 500 time-steps, at which point its viability is assessed. We repeated this procedure until a viable population was found, *i.e.*, with at least 500 viable individuals after the 500 time-steps. In this case, some organisms possess at least one pump to internalize *m*_*exo*_, and (because *m*_*exo*_ is not a prime number, see the description of the score function above) one enzyme to transform *m*_*exo*_ into a prime number, thereby producing an “essential metabolite”. Up to a few hundred trials were usually needed to find a viable population, which was then used to seed the evolutionary run. Each evolutionary run was seeded with a different viable population. These organisms grow on the primary resource and start to release by-products (mostly at death), hence modifying their environment. Populations evolved in two different environments:

1. The **periodic environment**, in which the resource dynamics of the LTEE [15] was mimicked. The environment was periodically refreshed by removing all the external metabolites and introducing *m*_*exo*_ at concentration *f*_*in*_ = 10.0 ACU per grid spot. Internal metabolites were not affected by the refresh event. The refresh period was Δ*t* = 333 time-steps. We call a “cycle” this time interval between two environmental resets. The value of Δ*t* was calibrated to let the organisms live for approximately 7 generations per cycle, as in the LTEE. Within each cycle, the metabolites in the environment were conserved (*D*_*g*_ = 0 per time-step). Note that we mimicked the resource dynamics of the LTEE but not the 1% population subsampling occurring during serial transfers, because it would have implied transferring populations of 10 individuals or fewer. Such a low population size would have implied dramatic genetic drift and impeded adaptive evolution (in the LTEE, where the population size before sampling is very large, the 1% subsampling still leaves the population large enough to keep genetic drift reasonably low). To simulate subsampling, a significantly larger grid would have been needed, making the whole campaign impossible to compute in a reasonable time.
2. The **continuous environment**, in which the resource dynamics of a chemostat environment was mimicked. The medium was constantly provided with a small influx of the primary resource. All the external metabolites were slowly degraded. Specifically, at each time-step, a concentration Δ*f*_*in*_ = 0.03 ACU of *m*_*exo*_ was added in every grid spot, and external metabolites were degraded at rate *D*_*g*_ = 0.003 per time-step.

For each environment, 12 independent populations were propagated for 500,000 time-steps (approximately 50,000 generations). On the long-term, the quantity of resources available in the system was equivalent in both environments. The grid size is 32 × 32. Complementary experiments were also run in a randomized batch environment similar to the periodic environment except that the environment reset intervals followed a Poisson law of parameter Δ*t* = 333 time-steps instead of the exact regular period of 333 time-steps. The simulation parameters common to all the simulations are described in S1 Table.

#### Cross-feeding interactions

In order to detect the potential cross-feeding interactions in the population, the metabolic activity of each individual was evaluated at each time-step. For each organism, a “trophic profile” was computed from its metabolic network activity. The trophic profile is a binary sequence summarizing the uptake, production and release activity of an organism. The length of the binary string was defined by the largest metabolite tag present in the system at time *t*. For example, if an organism uptakes metabolite #4, produces #3 from #4 and releases #3, knowing that the largest metabolite tag in the whole grid is #5, then its profile is |00010|00100|00100|. We classified organisms in two trophic groups depending on their trophic profiles:

1. “Group A” pumps in *m*_*exo*_, and possibly other metabolites,
2. “Group B” pumps in group A by-products, and possibly other metabolites, but not the primary resource *m*_*exo*_.

A trophic group is considered an ecotype if the organisms of the group form a monophyletic cluster (see below).

#### Phylogenetic relationships

Phylogenetic relationships were exhaustively recorded during each simulation. Since organisms can only divide once per time-step, phylogenetic trees are binary trees. It was possible to recover the line of descent of any organism, and to compare the phylogenetic tree structure with the distribution of the trophic groups in the population. In particular, we can determine if groups A and B are monophyletic, and thus can be considered as ecotypes. To this aim, we computed a phylogenetic structure score (*PS* score) to identify the degree of monophyly of both groups. This phylogenetic structure score was defined as *PS* = |*f*_1_ − *f*_2_|, where *f*_1_ and *f*_2_ are the relative frequencies of group B in both subtrees rooted to the last common ancestor of the whole final population. A high *PS* value indicates a strong clustering of groups A and B in the phylogenetic tree, *i.e.*, that groups A and B are two different ecotypes. A low *PS* value indicates a random distribution or the absence of polymorphism.

### Sensitivity analysis

We tested variations of our parameters set (see S1 Table), by changing the death probability *p*_*death*_, the external metabolites diffusion rate, the mutation rates, the toxicity thresholds, the “migration rate” (a parameter controlling the fraction of exchanged pairs among all possible pairs of individuals), and the grid size. Details and results are described in S1 Appendix.

## Results

**Fig 2:**
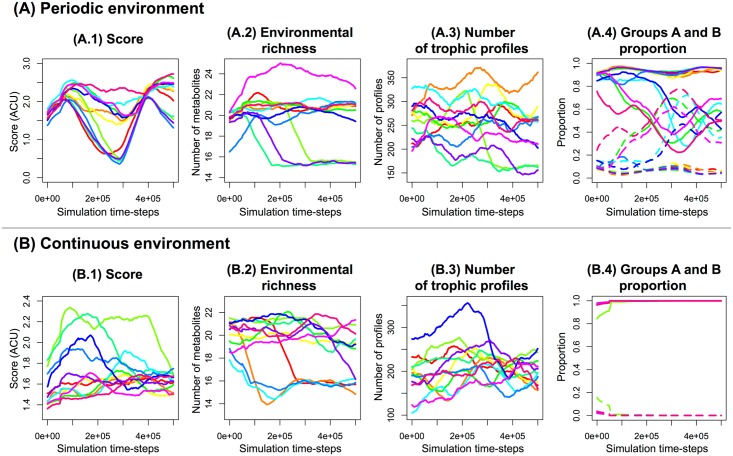
Evolution of typical variables. Evolution of the mean score (**A.1** and **B.1**), the environmental richness (the number of different metabolites present in the environment, **A.2** and **B.2**), the number of trophic profiles (**A.3** and **B.3**), and the proportion of organisms of group A or B are represented (**A.4** and **B.4**). **(A)** Evolution in the periodic environment. **(B)** Evolution in the continuous environment. In **A.4** and **B.4**, group A is represented in solid lines and group B in dashed lines.

First, the global evolutionary dynamics of the system can be analyzed by looking at main simulation statistics. The evolution of the mean score, the environmental richness (the number of different metabolites available in the environment), the number of trophic profiles, and the proportion of organisms of group A or B are represented in Fig 2. The score and the environmental richness were of the same order of magnitude in the continuous and the periodic environments, but they were more stable in the continuous environment. The number of trophic profiles showed no striking difference between the periodic and the continuous environment (Fig 2A.3 and 2B.3), indicating that polymorphism was common in both situations. However, the dynamics of groups A and B were completely different. In the periodic environment, groups coexisted, even if they showed long-term frequency variations (Fig 2A.4). In the continuous environment, group B quickly emerged too but also quickly disappeared in all cases (Fig 2B.4). Thus, even if the diversity of trophic profiles was similar in both environments, all profiles belonged to group A in the continuous environment. Hence, there was no group exclusively specialized on by-products in the continuous environment, while they were common in the periodic one.

### Impact of environmental dynamics on evolved genome and network organization

We compared the structure of both the genome and the metabolic network of final A organisms (after 500,000 time-steps) from the continuous and periodic environments of the main campaign (see above). We evaluated five variables: **(i)** the mean genome size, **(ii)** the mean amount of non-coding DNA, **(iii)** the mean number of enzyme coding units encoding the same metabolic reaction (the “metabolic redundancy”), **(iv)** the mean number of different essential metabolites pumped in (the “uptake diversity”), and **(v)** the mean number of different essential metabolites produced (the “production diversity”). For each measure, we performed a two-sample Wilcoxon test with a Bonferroni correction (*n* = 5).

As shown in Table 1, there was no significant variation in the amount of non-coding DNA and the uptake diversity. By contrast, genome size (resp. 227.47 and 346.24 units, p-value < 0.001/5) and metabolic redundancy (resp. 15.80 and 7.98 units, p-value < 0.001/5) were significantly lower in the periodic environment compared to the continuous environment. Moreover, the number of essential metabolites produced was significantly higher in the periodic environment than in the continuous environment (resp. 5.04 and 6.58 essential metabolites, p-value < 0.001/5). These differences are explained by selective pressures on the metabolic network. Indeed, organisms experienced a trade-off between maximizing their score (*i.e.*, maximizing the concentration of essential metabolites in their cytoplasm) and avoiding lethal toxicity thresholds. In the periodic environment, the external resource *m*_*exo*_ was introduced by bursts of 10.0 ACU at each serial transfer. Thus, to maximize the score without reaching toxicity thresholds, organisms must avoid specializing on a single essential metabolite and instead spread the toxicity by distributing metabolic fluxes in the production of several essential metabolites. In the continuous environment, the external resource was continuously provided at a lower concentration (0.03 ACU at each time-step). In this case, the selective pressure on toxicity was relaxed and the number of essential metabolites produced was significantly lower. Interestingly, metabolic fluxes were also adjusted by amplifying or deleting genes. Indeed, in the continuous environment, there were more copies of E (enzyme coding units) than in the periodic environment, while the production diversity was lower, meaning that those units were amplified in the continuous environment to maximize metabolic fluxes, thus increasing the genome size.

**Table 1:** Comparison of the structure of the genome and metabolic network structure of final A organisms evolved in the continuous and periodic environments.

Variable	Continuous env.	Periodic env.	Wilcoxon test	Units
Genome size	346.24 ± 12.98	227.47 ± 53.21	***	Genomic units
Non-coding DNA	5.69 ± 1.21	4.69 ± 1.54	-	Genomic units
Metabolic redundancy	15.80 ± 1.84	7.98 ± 2.08	***	Genomic units
Uptake diversity	3.48 ± 0.37	3.87 ± 1.41	-	Metabolites
Production diversity	5.04 ± 0.31	6.58 ± 0.93	***	Metabolites

Five variables were evaluated: **(i)** the mean genome size, **(ii)** the mean amount of non-coding DNA, **(iii)** the mean number of E encoding the same metabolic reaction (the “metabolic redundancy”), **(iv)** the mean number of different essential metabolites pumped in (the “uptake diversity”), and **(v)** the mean number of different essential metabolites produced (the “production diversity”). The standard deviation is also shown (mean ± sd.). For each measure, we performed a two-samples Wilcoxon test, with Bonferroni correction (*n* = 5).

**Fig 3:**
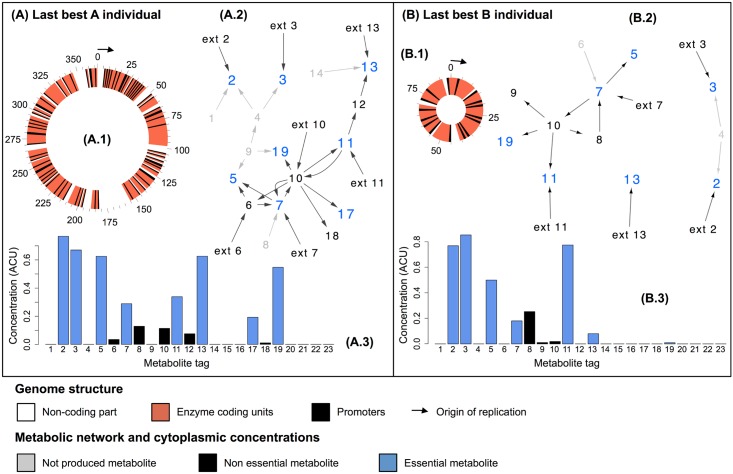
Final best individuals of groups A and B, from repetition 10 of the periodic environment. **(A)** Final best organism A. **(B)** Final best organism B. **(A.1, B.1)** The circular single-stranded genome. Non-functional regions are white, promoters black, E red, revealing numerous operons all along the genomes. **(A.2, B.2)** The metabolic network. Non essential and essential metabolites are colored in black and blue, respectively. Non-functional parts of the metabolic network (where fluxes are null) are shown in grey. **(A.3, B.3)** The internal metabolic concentrations (non essential metabolite concentrations: black. Essential metabolite concentrations: blue).

Fig 3 shows an example of organisms A and B evolved in the periodic environment after 500,000 time-steps (repetition 10). The final best individual of groups A (Fig 3A) and B (Fig 3B) are represented including their genome (Fig 3A.1 and 3B.1), metabolic network (Fig 3A.2 and 3B.2) and internal metabolic concentrations (Fig 3A.3 and 3B.3). The metabolic network of organism A was structured around *m*_*exo*_ (this metabolite being a hub in the network), even if the organism also fed on some by-products. Organism B’s metabolic network was less complex, and indicates that the organism mostly grew on A-secreted products. Most parts of both genomes were coding enzymes, revealing large operons all along the genomes.

### Relationship between ecology and phylogeny

For each simulation, we analyzed the final phylogenetic tree and compared it to the distribution of groups A and B. All the phylogenetic trees are represented in S1 Fig. Leaves are colored depending on their trophic group (group A in blue, group B in green). The structure of the trees was strongly related to the type of environment. In the periodic environment (S1 Fig), 5 phylogenetic trees among 12 (repetitions 1, 3, 7, 9 and 10) showed two well-separated clusters, each belonging to one ecological group. In these repetitions, two ecotypes evolved separately and remained stable on the long-term, showing that a stable cross-feeding interaction evolved. In the seven other cases, trees were less deep, had no well separated clusters, and no clear correlation between ecological groups and phylogenetic structure was observed. In the continuous environment (S1 Fig), trees were much shorter than in the periodic environment. Group A went to fixation in all repetitions. Then, while polymorphism and cross-feeding existed at a similar level in both periodic and continuous environments (Fig 2A.3 and 2B.3), this polymorphism was not stable in the continuous environment.

### Evolution of phylogenetic structure and trophic groups

**Fig 4:**
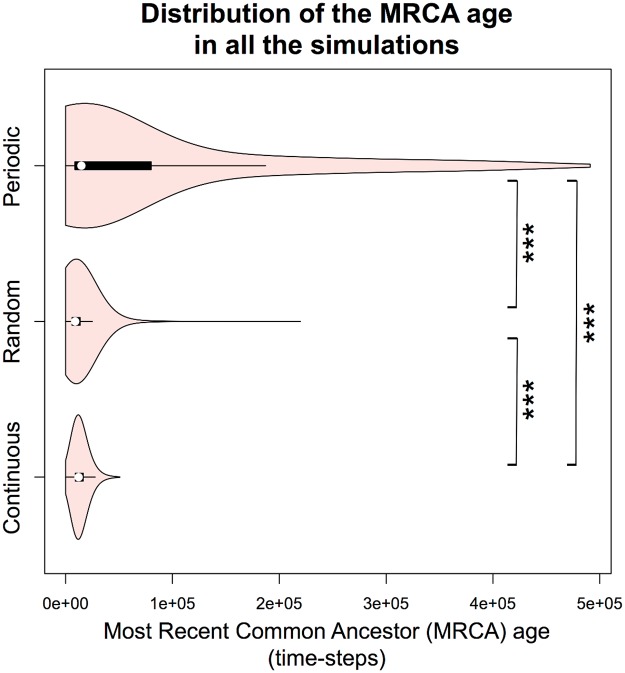
Distribution of the Most Recent Common Ancestor age in all the simulations. For each environment, we computed the distribution across repetitions of the Most Recent Common Ancestor (MRCA) age, for each simulation time-step. All pairwise Student tests are significant, with Bonferroni correction (p-value < 0.001/3).

To get more insight into the evolutionary dynamics, we computed the distribution of the Most Recent Common Ancestor (MRCA) age at each time-step for all the simulations. The MRCA age reflects the stability of the polymorphism in a population. As shown in Fig 4, distributions confirmed that the deepest trees evolved in the periodic environment, with a mean MRCA age of 71,004 time-steps, and a large distribution tail (some trees having almost the same depth as the total simulation time—500,000 time-steps). By contrast, the mean MRCA age is only 13,524 time-steps in the continuous environment and 11,684 time-steps in the complementary experiment with randomized refresh. This result indicates that environmental variations must be regular to favor stable cross-feeding interactions. The evolution of MRCA age during simulations is also represented in S2 Fig, for the three types of environment. This figure gives a better idea of the evolutionary dynamics of the phylogenetic trees. It shows that the MRCA age regularly collapsed in the random and continuous environments, but was still increasing for some simulations in the periodic environment.

**Fig 5:**
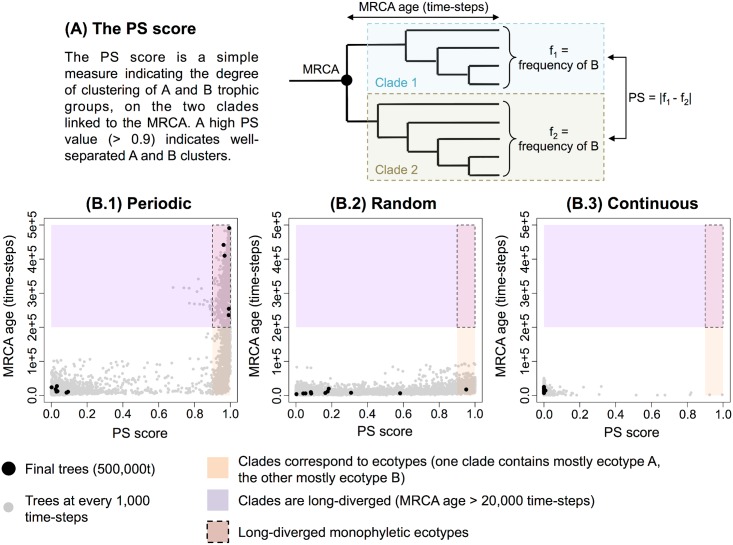
Phylogenetic structure score against the MRCA age. **(A)** The PS score. The PS score is a measure indicating the degree of clustering of A and B trophic groups, on the two clades linked to the MRCA. A high PS value (> 0.9) indicates well separated A and B clusters. **(B)** For each environment, the PS score is plotted against the MRCA age every 1,000 time-steps, for all repetitions, with the points corresponding to the final trees (at 500,000 time-steps) colored in black. Purple area: long-diverged clades (MRCA age higher than 200,000 time-steps). Orange area: clades corresponding to ecotypes (PS score > 0.9). Intersection (inside dashed borders): long-diverged monophyletic ecotypes. **(B.1)** Periodic environment. **(B.2)** Random environment. **(B.3)** Continuous environment.

We then compared the phylogenetic structure with the distribution of groups A and B on tree leaves by computing the phylogenetic structure score *PS* (Fig 5A). In Fig 5B, this *PS* score is plotted against the MRCA age every 1,000 time-steps for all the repetitions (grey points). Points corresponding to the end of each simulation are colored in black. On each plot, three areas are identified: the purple area indicates long-diverged clades (MRCA age higher than 200,000 time-steps), the orange area indicates when clades correspond to ecotypes (PS score > 0.9), and the intersection of the previous two areas (inside dashed borders) indicates long-diverged monophyletic ecotypes. In the periodic environment (Fig 5B.1), the deepest trees were also the most structured, with two well separated monophyletic ecotypes A and B. In the random environment (Fig 5B.2), the situation was contrasted, with a large distribution of the *PS* score, ranging from monomorphic trees (A or B groups being fixed), to polymorphic trees. However, the MRCA age was very short compared to the periodic environment, revealing the instability of the phylogenetic structure. Note that the random environment is the only one where we observed a population extinction (1 out of 12). In the continuous environment (Fig 5B.3), the population was mostly monomorphic (group A being fixed), with short MRCA ages.

To evaluate the robustness of these results to the variation of main simulation parameters, we performed a sensitivity analysis. The results are presented in details in S1 Appendix. Even if some parameters were more sensitive than others (*e.g.*, the death probability and the toxicity thresholds, discussed in S1 Appendix), this analysis revealed that our results are robust. In the continuous environment, no single simulation evolved a stable A/B cross-feeding in the whole analysis. Moreover, when the diffusion rate was infinite, or when the population was perfectly mixed (all locations being randomized at each time-step), almost all repetitions (80% in infinite diffusion conditions, 100% in well-mixed conditions) evolved a stable A/B cross-feeding in the periodic environment (see S1 Appendix). This result is in agreement with previous studies showing that the spatial structure may affect polymorphism [39, 40].

These results confirmed that the periodic environment strongly favored the evolution of stable cross-feeding interactions, in contrast to the random and continuous environments, in apparent contradiction with the results of wet experiments in chemostat, and we will discuss this point below.

### Evolution of trophic profiles

**Fig 6:**
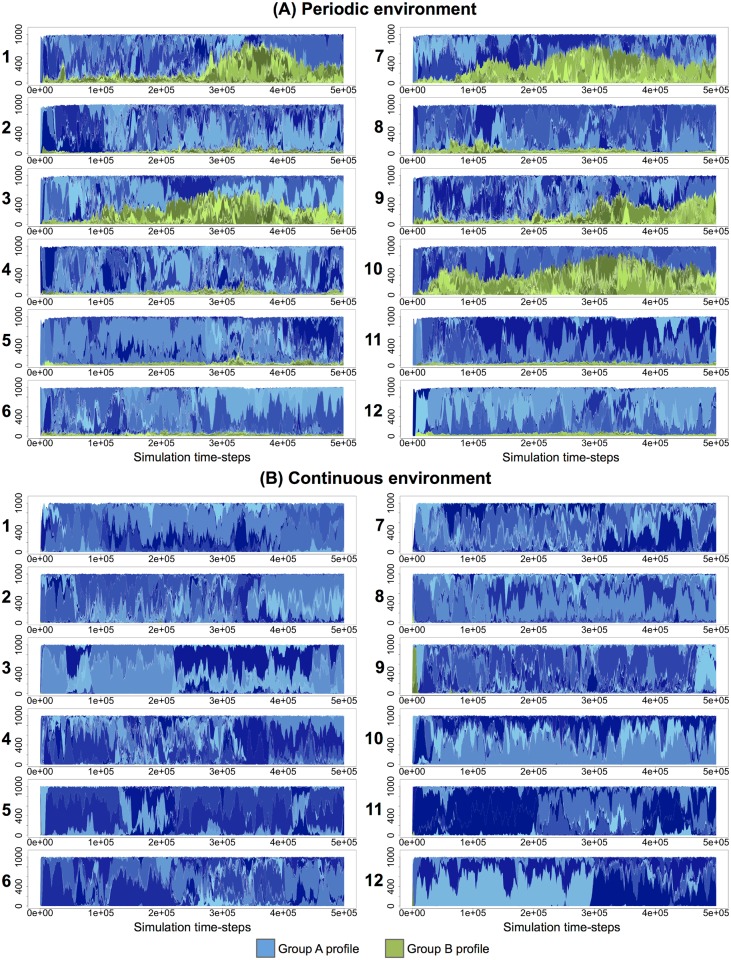
Evolution of trophic profiles in the population for the continuous and periodic environments. Trophic profiles gather organisms that own the exact same metabolic activity (see Methods). Blue and green profiles belong to trophic groups A and B, respectively. **(A)** Continuous environment simulations. **(B)** Periodic environment simulations.

We then recovered the proportion of trophic profiles over time (at every 1,000 time-steps) in all the simulations of the periodic and continuous environments (Fig 6A and 6B, respectively). Trophic profiles belonging to groups A and B are colored in shades of blue and green, respectively. Those figures show that evolution in the model was ruled by periodic selection in a highly polymorphic population. This polymorphism was mainly due to competition for resources, with the organisms constantly competing for the primary resource but also the by-products available in the environment.

However, in the periodic environment (Fig 6A), trophic profiles from groups A and B coexisted over time, with periodic selection events occurring independently in both groups. This dynamics is typical from multiple niche selection, where beneficial mutations do not spread in all the population owing to competitive exclusion, but are confined in one specific niche. In the continuous environment (Fig 6B), group A was predominant in all simulations, periodic selection affecting the whole population. In these conditions, the level of cross-feeding was maintained but the interactions were not stable (as shown by Fig 2A.3 and 2B.3).

These results reinforce the fact that stable cross-feeding interactions were only possible in the periodic environment. Specifically, in the periodic environment, evolution was driven by multiple niche selection, with periodic selection events independently occurring in ecotypes A and B. On the opposite, in the continuous environment, evolution was driven by periodic selection and competitive exclusion, indicating that there was less opportunity for niche construction.

### Ecological dynamics in the periodic environment

Comparative analysis of phylogenetic structure in the different environments revealed that the periodic environment especially favored the evolution of stable cross-feeding interactions, leading to two monophyletic ecotypes A and B in 5 of 12 repetitions, with the ecotype A feeding on the primary resource and possibly on some by-products, while ecotype B consumed by-products. In the LTEE, it has been shown that the coexistence of S and L ecotypes is driven by negative frequency-dependent interactions [12, 13]. We analyzed in details the 5 populations to see whether the stable cross-feeding interactions were comparable to the S/L interaction.

#### Mutational history of ecotypes A and B

**Fig 7:**
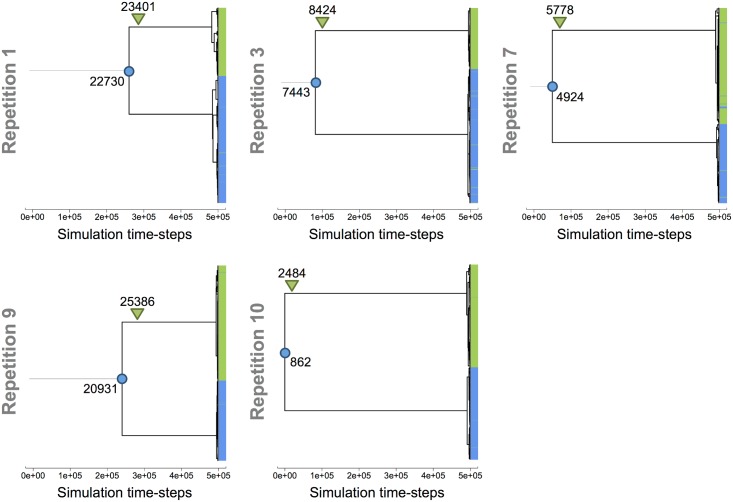
Analysis of the adaptive diversification event leading to the monophyletic ecotypes A and B. In the phylogenetic trees of final evolved populations, the colored circles indicate the trophic group and the generation of the common ancestor. The colored triangles indicate the generation when one monophyletic ecotype moved from one trophic group to the other (*i.e.*, losing or gaining pumps to feed on external nutrient). Group A (blue) grows on the primary resource and possibly on by-products. Group B (green) exclusively grows on by-products.

In the 5 populations that evolved a stable cross-feeding, we recovered the mutational history of the lineages of ecotypes A and B. Final phylogenetic trees of the 5 populations are represented in Fig 7. For each tree, the trophic group of the MRCA, as well as the generation at which one of the monophyletic ecotypes switched from the ancestor group to the other one (*i.e.*, when one ecotype lost or gained inflowing pumps for the primary resource), are shown. In all 5 populations, the same pattern emerged: the population was primarily of group A, but niche construction on by-products resulted in adaptive diversification, with one ecotype strongly specializing on by-products, such that it lost the ability to uptake the primary resource.

Interestingly, in all simulations, the loss of this ability was not the source of the adaptive diversification. The diversification event occurred a few hundreds of generations *before* the loss of the pump provoking the change of trophic group. In the LTEE, the S ecotype specialized on acetate, but was still able to grow on glucose. However, recent work has shown that while the S ecotype improved its ability to grow on acetate since the diversification event, it was not the case on glucose, presaging a possible complete loss of its ability to grow on glucose in the longer term [16]. Conversely, the L ecotype improved its ability to grow on glucose, but not on acetate, also presaging a loss of ability to grow on acetate at a longer term.

#### Ecotype B frequency-dependent fitness in short term competition experiments

**Fig 8:**
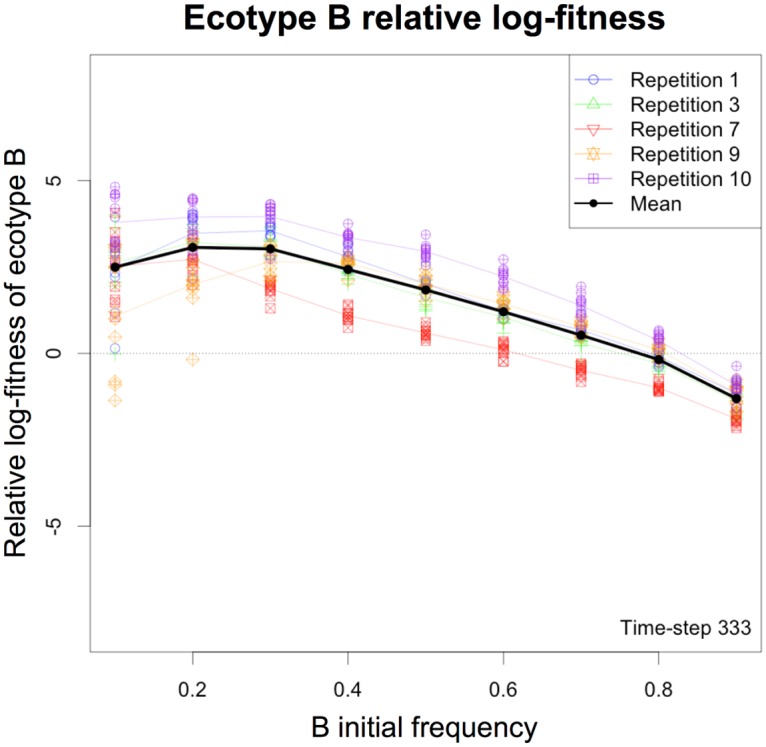
Frequency-dependent relative fitness in short-term competition experiments. The frequency-dependent fitness was computed using log-fitness [25, 41] in short term competition experiments, starting with different initial frequencies of B ecotype. For each of the 5 populations that evolved monophyletic ecotypes at the end of the simulations, 10 repetitions were run per initial frequency of B (0.1, 0.2, 0.3, 0.4, 0.5, 0.6, 0.7, 0.8 and 0.9). The global mean frequency-dependent fitness is represented in black. Mean fitness per population is shown in shaded colors. Each individual experiment is plotted in shaded color dots, related to their mean color.

To test whether ecotypes A and B coexistence is maintained by negative frequency-dependent interactions, we performed short term competition experiments with the 5 populations that evolved a stable cross-feeding interaction at the end of the simulations in the periodic environment (repetitions 1, 3, 7, 9 and 10). Initial populations were seeded at 9 different initial frequencies of B (0.1, 0.2, 0.3, 0.4, 0.5, 0.6, 0.7, 0.8 and 0.9—each with 10 repetitions) and were propagated in the same periodic environment during 1 cycle (*i.e.*, 333 time-steps). Then, we computed the log-fitness [41] of ecotype B, taking into account its initial frequency and its frequency at the end of the first cycle. Fig 8 demonstrates that the ecotypes A and B interaction was frequency-dependent, the ecotype B being favored when initially rare, and penalized when initially abundant. Since the external conditions varied during the seasons, the B organisms were not favored during the whole cycle. S1 Video shows the variation of B relative fitness over the 333 time-steps of the first cycle, at a full temporal resolution. This video shows the establishment of the negative frequency-dependent interaction along the cycle, and reveals that the relative fitness of B was initially negative at all initial frequencies. Indeed, at each cycle, B ecotype growth was delayed compared to A ecotype, the former growing on by-products during the second season, while the latter grew on fresh primary resource during the first season. At low initial frequencies of B, their small number can randomly lead to their extinction, thus artificially reducing its mean relative fitness. Those results are in full agreement with the LTEE [11, 25].

#### Convergence to an oscillatory dynamics

**Fig 9:**
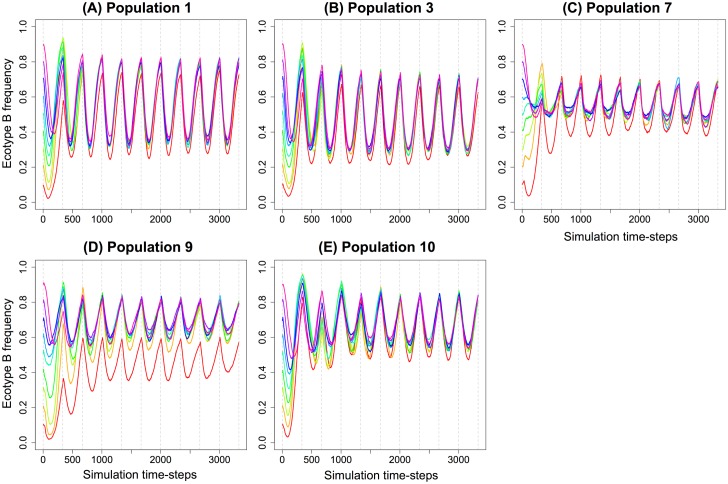
Convergence to an oscillatory dynamics over 10 serial transfer cycles. Ecotype B is advantaged when rare, but is penalized when initially common, leading to a balanced polymorphism. Nine different initial frequencies of B have been tested (0.1, 0.2, 0.3, 0.4, 0.5, 0.6, 0.7, 0.8 and 0.9). Each trajectory is the mean of the B frequency among the 10 repetitions of each of the 5 populations. **(A)** Population 1. **(B)** Population 3. **(C)** Population 7. **(D)** Population 9. **(E)** Population 10.

Owing to their negative frequency-dependent interaction, the relative frequencies of ecotypes A and B should stabilize over time, as in the LTEE [11]. We extended the previous competition experiments to 10 cycles and recorded the A and B proportions at each time-step (Fig 9). Trajectories show that at all initial frequencies of B, a stable oscillatory dynamics was reached for each repetition (Fig 9A for repetition 1, Fig 9B for rep. 3, Fig 9C for rep. 7, Fig 9D for rep. 9 and Fig 9E for rep. 10). The observed variability was due to contingent evolutionary differences between the 5 populations, and to a sampling effect when the initial frequency of B was low. Here again we observed exactly the dynamics observed in the LTEE [11, 25], even if the small population size artificially increased the oscillatory dynamics.

### Stability of the A/B cross-feeding interactions when transferred in the continuous environment

In the continuous environment, no stable cross-feeding interaction evolved. This result is in apparent contradiction with wet experiments during which *E. coli* populations evolved in a continuous culture with glucose as a single limiting carbon source [7, 8, 10]. In those experiments, cross-feeding interactions emerged after a few hundreds of generations. Nonetheless, our results showed that cross-feeding interactions quickly emerged in the continuous environment, but these interactions were not stable (Fig 2).

To test whether a population with two stable A and B ecotypes (evolved in the periodic environment) could persist when placed in the continuous one, we let populations from the periodic environment evolve in chemostat-like environments for 500,000 time-steps. The 5 populations that evolved a stable cross-feeding in the periodic environment were propagated in a continuous environment at two different stages of their evolution: **(i)** just after adaptive diversification (early populations, see Fig 7), **(ii)** and at the end of the simulations (late populations, after 500,000 time-steps). As a control, these populations were also propagated in the periodic environment. For each population, 10 repetitions were run in each environment. Then, we evaluated the stability of the A/B cross-feeding interaction by counting the number of simulations where the interaction persisted, the number of simulations where the interaction failed, and the time before interaction failure.

**Fig 10:**
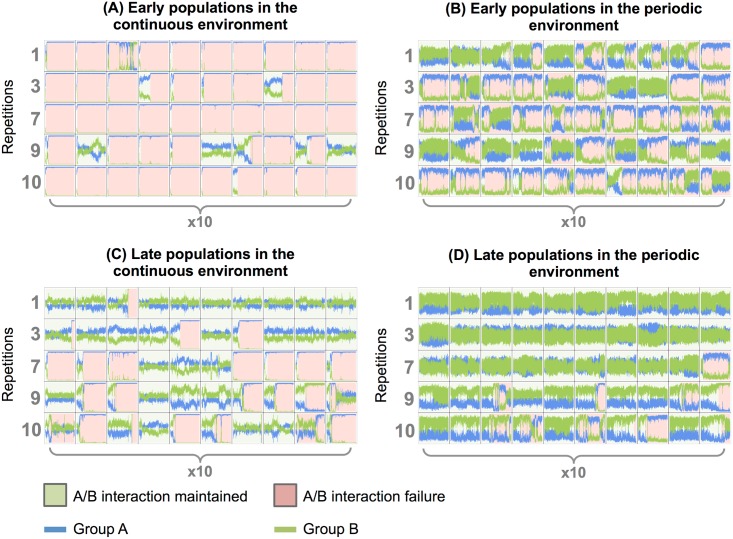
Stability of the A/B interaction evolved in the periodic environment, when placed in the continuous one. Early populations were transferred just after adaptive diversification. Late populations were transferred at the end of the simulations (500,000 time-steps). For each repetition that evolved two ecotypes A and B (rep. 1, 3, 7, 9 and 10), 10 repetitions of 500,000 time-steps were run. The stable polymorphism was considered to be lost if the MRCA age changed, indicating that one of the two monophyletic groups was outcompeted. In this case, the simulation is colored in green, and red before and after this event, respectively (simulations where the A/B interaction was maintained during the whole experiment are fully green). **(A)** Early populations transferred in the continuous environment. **(B)** Early populations transferred in the periodic environment. **(C)** Late populations transferred in the continuous environment. **(D)** Late populations transferred in the periodic environment.

The proportion of simulations where the interaction persisted are displayed in Table 2 for the continuous environment, and in Table 3 for the periodic environment. The evolution of the proportions of groups A and B is also shown in Fig 10A and 10C for the continuous environment (early and late populations, respectively), and in Fig 10B and 10D for the periodic environment (early and late populations, respectively). First, Table 2 shows that, for early populations in the continuous environment, the interaction was not robust and persisted in only 6% of the assays. For late populations in the continuous environment, the interaction was more robust, as the polymorphism persisted in 50% of the assays. For early populations in the periodic environment, the interaction was also not robust (the interaction persisted in only 18% of the assays), even if more populations maintained the interaction than in the continuous environment. This low percentage is probably due to the experimental protocol: populations were transferred in a periodic environment at the beginning of a new cycle, whatever the previous seasonal context of the population. The interaction was then destabilized while the diversification event was still recent, leading to a high probability to loose the interaction (a situation similar to what occurred in the random environments). However, for late populations in the periodic environment, most assays kept the polymorphism stable (the interaction persisted in 78% of the assays), indicating that seasonality is of primary importance to stabilize the interaction.

**Table 2:** Proportion of assays where polymorphism persisted in chemostat conditions.

	Pop 1	Pop 3	Pop 7	Pop 9	Pop 10
Early populations	0%	0%	0%	30%	0%
Late populations	90%	70%	30%	40%	20%

For each population, 10 assays were simulated. The stable polymorphism was considered to be lost if the MRCA age changed, indicating that one of the two monophyletic groups was outcompeted.

**Table 3:** Proportion of assays where polymorphism persisted in batch conditions.

	Pop 1	Pop 3	Pop 7	Pop 9	Pop 10
Early populations	30%	20%	0%	40%	0%
Late populations	100%	100%	90%	60%	40%

For each population, 10 assays were simulated. The stable polymorphism was considered to be lost if the MRCA age changed, indicating that one of the two monophyletic groups was outcompeted.

**Fig 11:**
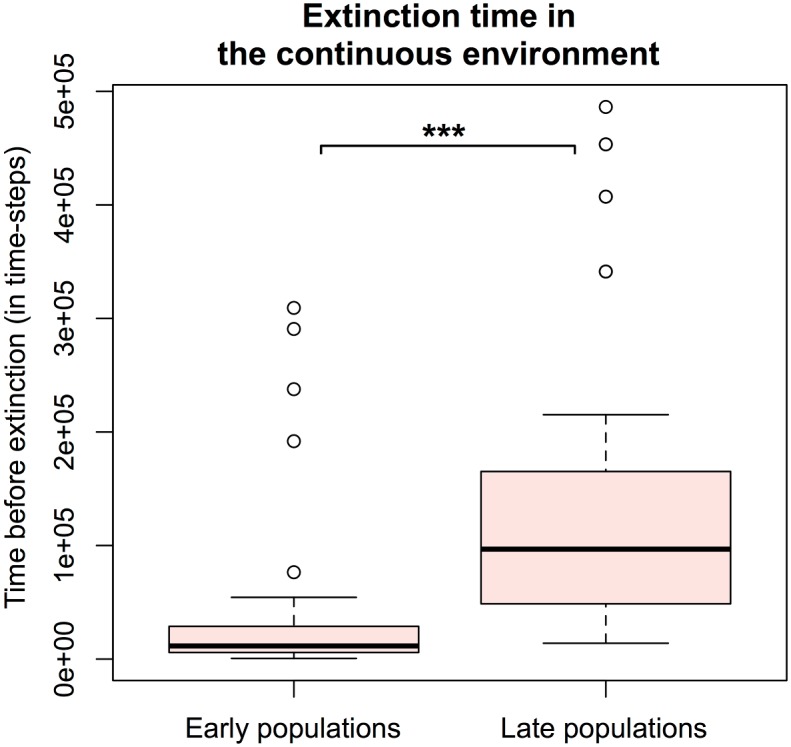
Time before A/B interaction failure in the continuous environment. The time (in time-steps) is measured for all competition experiments in the continuous environment (50 simulations) where the interaction failed. Persistent interactions are not considered here. Early populations loose the interaction significantly earlier than the late ones (Student test is significant with a p-value < 0.001).

Fig 11 shows the distribution of the time before A/B interaction failure for early and late populations in the continuous environment. Late populations were much more robust, since extinctions happened significantly later (with a mean of 142,291.2 time-steps) than for early populations (with a mean of 37,173.68 time-steps). Student test gives a p-value < 0.001.

#### Vulnerability of ecotype B to A fast-growing mutants when transferred in the continuous environment

In order to understand why the A/B interaction failed in half of the continuous environment experiments (50% of the assays in the late populations), and why the A/B interaction failures implied the extinction of ecotype B in most cases (80% of the failures in the late populations), we studied in details the evolution of digital organisms in this environment.

For each late population propagated in the continuous environment (Fig 10C), we compared the initial A ecotype to the final A ecotype (after 500,000 time-steps of evolution in the continuous environment). We performed the same genomic and metabolic analyse as in section “Impact of environmental resource dynamics on evolved genome and network organization”. Table 4 shows that A organisms **(i)** significantly increased their genome size (from 189.99 to 269.73 units, p-value < 0.001/5), their mean metabolic redundancy (from 6.69 to 10.86 units, p-value < 0.001/5) and mean uptake diversity (from 1.17 to 1.69 metabolites, p-value < 0.001/5), and **(ii)** significantly decreased their mean production diversity (from 7.14 to 5.66 metabolites, p-value < 0.001/5), when they evolved in the continuous environment for 500,000 time-steps. Indeed, the relaxation of selective pressures to maintain concentrations under the lethal toxicity thresholds led to a restructuring of A organisms towards a genome and metabolic network well-adapted to continuous conditions (see Table 1). This modification of ecotype A phenotypes impaired the negative frequency-dependent interaction between the A and B ecotypes: since B organisms consumed A-secreted by-products, the reduction of the production diversity of A organisms led to the extinction of ecotype B in half of the assays.

**Table 4:** Comparison of the genome and metabolic network structure of initial and final ecotype A, when transferred in the continuous environment.

Variable	Initial pop.	Final pop. (500,000t)	Wilcoxon test	Units
Genome size	189.99 ± 49.85	269.73 ± 96.69	***	Genome units
Non-coding DNA	4.69 ± 2.10	5.18 ± 2.01	-	Genome units
Metabolic redundancy	6.69 ± 1.46	10.86 ± 4.81	***	Genome units
Uptake diversity	1.17 ± 0.54	1.69 ± 1.13	***	Metabolites
Production diversity	7.14 ± 0.73	5.66 ± 1.78	***	Metabolites

Five variables were evaluated: **(i)** the mean genome size, **(ii)** the mean amount of non-coding DNA, **(iii)** the mean number of E encoding the same metabolic reaction (the “metabolic redundancy”), **(iv)** the mean number of different essential metabolites pumped in (the “uptake diversity”), and **(v)** the mean number of different essential metabolites produced (the “production diversity”). The standard deviation is also shown (mean ± sd.). For each measure, we performed a two-samples Wilcoxon test, with Bonferroni correction (*n* = 5).

**Fig 12:**
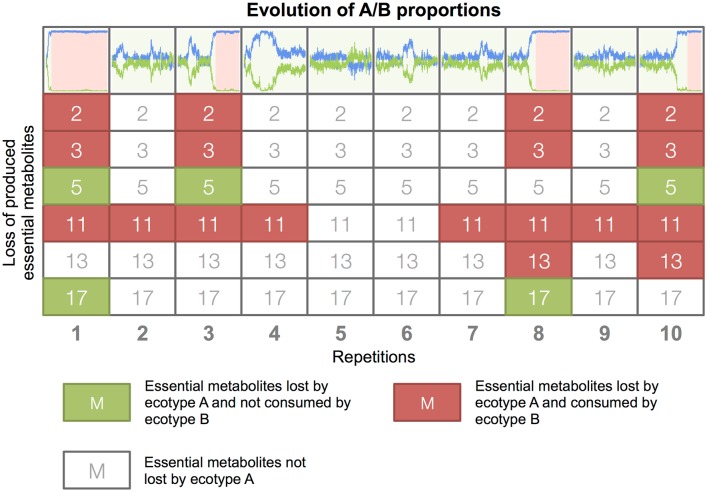
Loss of essential metabolites production of ecotype A organisms in the 10 repetitions of the late population 3 in the continuous environment assays. The 10 repetitions of the population 3 are displayed. The 8 essential metabolites (2, 3, 5, 7, 11, 13, 17, and 23) that were produced by ecotype A organisms at the beginning of the assays are represented vertically for each repetition. Colored metabolites indicate a production loss. Essential metabolites that are consumed by ecotype B organisms are colored in red, the others in green. At the top, the evolution of groups A and B proportions is represented in green when the A/B interaction persisted and in red when the interaction failed. In all simulations where A ceased to produce a metabolite pumped-in by B, B went to extinction.

To exemplify these statistical results, we studied in details the evolution of ecotypes A and B in the 10 repetitions of the late population 3, when propagated in the continuous environment (Fig 10C.3). At the beginning of the assays, ecotypes A and B interacted through a negative frequency-dependent cross-feeding: ecotype A organisms produced essential metabolites 2, 3, 5, 7, 11, 13, 17 and 23; ecotype B organisms consumed metabolites 2, 3, 5 and 7 (all secreted by ecotype A organisms). We evaluated the evolution of the 8 essential metabolites that were produced by ecotype A organisms at the beginning of the assays (Fig 12). Ecotype A organisms reduced their production of essential metabolites in all assays. However, when ecotype A organisms stopped producing metabolites 2, 3, 5 and/or 7, ecotype B organisms systematically went to extinction. On the opposite, when ecotype A organisms stopped producing metabolites 17 and/or 23 (but maintained the production of 2, 3, 5 and 7), ecotype B was not affected. These results confirm the mechanism of B extinction: when placed in continuous conditions, ecotype A organisms reorganized their metabolism and produced fewer essential metabolites. Now, while doing so they may stop producing metabolites that were necessary for the survival of ecotype B organisms, leading to their extinction.

#### The robustness of the A/B interaction in late populations is explained by character displacement and niche specialization

In order to understand why the interaction between A and B ecotypes was more robust in late than early populations, we compared the genomic and metabolic structures of early and late populations, independently for A and B ecotypes. We performed the same statistical tests as in Tables 1 and 4 (two-samples Wilcoxon test with Bonferroni correction of *n* = 5). The results are presented in Table 5. In both ecotypes, the genome size, amount of non-coding DNA and metabolic redundancy were significantly reduced. However, if ecotype A significantly reduced its uptake diversity and increased its production diversity, ecotype B evolved in an opposite way (*i.e.*, ecotype B significantly increased its uptake diversity and reduced its production diversity). The traits of ecotypes A and B diverged: **(i)** ecotype A strongly specialized on *m*_*exo*_ (with a mean uptake diversity of 1.17 metabolites), and optimized metabolic fluxes according to the trade-off between avoiding lethal toxicity thresholds and maximizing the score (as explained above, this selective pressure resulted in reduced genome size and metabolic redundancy, and increased production diversity). **(ii)** Ecotype B specialized on by-products by increasing the uptake diversity and reducing the production diversity. This demonstrates that ecotypes A and B specialized to their own niches, and that ecotypes A and B traits diverged by character displacement, in complete agreement with the LTEE [16, 34].

**Table 5:** Comparison of the genome and metabolic network structure of initial ecotypes A and B in the early and late populations.

Variable	Early pop.	Late pop.	Wilcoxon test	Units
A genome size	257.09 ± 42.31	189.99 ± 49.85	***	Genome units
A non-coding DNA	6.37 ± 1.14	4.69 ± 2.10	**	Genome units
A metabolic redundancy	8.20 ± 0.83	6.69 ± 1.46	***	Genome units
A uptake diversity	3.12 ± 0.51	1.17 ± 0.54	***	Metabolites
A production diversity	6.82 ± 0.12	7.14 ± 0.73	*	Metabolites
B genome size	274.93 ± 31.98	203.14 ± 59.59	***	Genome units
B non-coding DNA	9.70 ± 8.21	4.20 ± 2.05	***	Genome units
B metabolic redundancy	9.86 ± 2.48	8.04 ± 2.54	**	Genome units
B uptake diversity	3.74 ± 0.76	4.59 ± 0.84	***	Metabolites
B production diversity	6.26 ± 0.70	5.85 ± 0.57	**	Metabolites

For each ecotype, five variables were evaluated: **(i)** the mean genome size, **(ii)** the mean amount of non-coding DNA, **(iii)** the mean number of E encoding the same metabolic reaction (the “metabolic redundancy”), **(iv)** the mean number of different essential metabolites pumped in (the “uptake diversity”), and **(v)** the mean number of different essential metabolites produced (the “production diversity”). The standard deviation is also shown (mean ± sd.). For each measure, we performed a two-sample Wilcoxon test with Bonferroni correction (*n* = 5).

Character displacement explained the apparent robustness of the A/B interaction in late populations. Indeed, niche specialization led A organisms to specialize on *m*_*exo*_ and increase their production diversity. On the other hand, B organisms specialized on a large number of by-products. However, character displacement and niche specialization was stronger in late than early populations. For this reason, late A organisms needed more mutations (and then more evolution time) to adapt to continuous conditions (*i.e.*, reducing the production diversity and increasing metabolic redundancy, see Table 1) than early A organisms. Thus, B organisms were slower out-competed in late than early populations.

To assess those conclusions, we studied in details the evolution of ecotypes A and B in the 10 repetitions of the early population 3 (Fig 10A.3), in the exact same way than in Fig 12. The result is available on S3 Fig, and shows that A organisms from early populations reduced their production diversity (mean of 3.4 metabolites) more than A organisms in late populations (mean of 2.0 metabolites). Indeed, A organisms were less specialized and thus needed less time to adapt to the continuous conditions, thereby favoring the extinction of ecotype B.

The fact that beneficial mutations from ecotype A spread all over the population in the continuous environment (Fig 4) indicates that competitive exclusion occurred. In this case, according to [14], A and B groups cannot be considered as separate ecotypes in the continuous environment, although the same organisms were separate ecotypes in the periodic environment. Note that in 5 assays over all experiments, ecotype B fixed in the population (1 assay for early populations in the continuous environment, 3 assays for late populations in the continuous environment, and 1 assay for late populations in the periodic environment). When ecotype B invaded the population, by-products were not produced anymore by ecotype A, therefore dooming the whole population to extinction (as exemplified in repetition 3 of late population 1).

Hence, the stability of the A/B cross-feeding interaction in the continuous environment relied on the evolutionary time elapsed in the periodic environment since adaptive diversification. This shows that the co-evolution of ecotypes A and B in the periodic environment strengthened their interaction, meaning that niche specialization stabilized the cross-feeding and fostered a robust negative frequency-dependence. However, even if the cross-feeding interaction seemed stable over few thousands of generations, in the continuous environment a beneficial mutation in ecotype A lineage can lead to the extinction of ecotype B lineage. Therefore, the stability of the A/B polymorphism in the periodic environment did not rely only on their cross-feeding interaction, but also on the seasonality of the environment.

## Discussion

Using *in silico* experimental evolution, we have shown that the long-term maintenance of cross-feeding interactions is favored in a seasonal environment, where the environment is reset and primary resource is supplied at regular intervals. In this environment, 5 simulations over 12 evolved a stable cross-feeding interaction at the end of the simulations, with two monophyletic ecotypes coexisting via a negative frequency-dependent interaction. At each cycle, ecotype A grows during the first season, feeding on the primary resource and releasing by-products, while ecotype B exclusively feeds on by-products during the second season. The stable coexistence of ecotypes A and B is then based on niche construction, followed by a negative frequency-dependent interaction, as the S and L ecotypes in the LTEE. According to our model, batch culture experiments seem to especially favor the evolution of stable cross-feeding polymorphisms owing to the cyclic nature of the environment that generates the conditions for the existence of at least two stable seasons: a first season is externally generated by the cyclic mechanism (thus being intrinsically stable) while the second one is generated by the replacement of the exogeneously-provided nutrient by the secreted by-products through a mechanism of niche construction.

In the continuous environment, where the primary resource is constantly provided (like in a chemostat), cross-feeding interactions emerged, but were not stable because of competitive exclusion. In this case, organisms enriched their environment via their metabolic activity, such that mutants were temporarily able to feed on by-products. But the absence of seasonality precludes any possibility for the stabilization of cross-feeding interactions.

Our multi-scale model allowed us to investigate the impact of resource dynamics on the organization of genome (*e.g.*, gene amplification) and of the metabolic network. It also allowed us to dissect the precise mechanism behind the evolved robustness of the cross-feeding interaction. We demonstrated that those results are robust to model parameter variation. Indeed, stable cross-feeding interactions emerged in the periodic environment for a wide range of parameter values, including well-mixed populations and infinite diffusion rate, while they never appeared in the continuous environment, thus reinforcing our conclusions (S1 Appendix).

Previous wet experiments in chemostat demonstrated the emergence of cross-feeding interactions [7, 8, 10]. In those experiments, *E. coli* populations have been propagated in a chemostat with glucose as a single limiting carbon source for at most 1,900 generations. When isolated and evolved together in competition experiments, the different mutants identified to contribute to the cross-feeding interactions reached a stable equilibrium owing to frequency-dependent interactions [8]. Several reasons were invoked to explain why cross-feeding interactions could be stable in chemostat, despite the competitive exclusion principle. According to Pfeiffer and Bonhoeffer [18], cross-feeding may evolve in microbial populations as a consequence of the maximization of ATP production, and the minimization of enzyme concentrations and intermediate products. Those constraints may hinder the emergence of mutants completely degrading glucose (or uptaking glucose and acetate), and outcompeting other cells by competitive exclusion. In our model, organisms do not need to explicitly produce energy carriers. However, competition for resources, toxicity thresholds and division impose metabolic flux optimization. Based on the same conclusions, Doebeli [23] also suggested that this trade-off between uptake efficiency on the primary and the secondary resources should favor the emergence of cross-feeding polymorphism in chemostat but not in batch culture, because in a chemostat, by-products are more abundant and constantly provided. However, the limit of this model is that the rate of by-product production did not rely on the rate of primary resource consumption. Besides, a more recent theoretical work concluded that, in a continuous and well-mixed environment, the diversity of cross-feeding polymorphism was negatively correlated with primary resource abundance [32].

Our results shed a new light on this question. First, in our model, cross-feeding polymorphisms emerged both in the periodic and continuous environments. However the stabilization of the cross-feeding interactions was favored in the periodic environment, leading to the evolution of specialized ecotypes. Cohan [14] defined an ecotype as an independent monophyletic cluster occupying a specific ecological niche. Ecotypes are at the heart of the bacterial species concept: what makes the genetic cohesion of an asexual bacterial species is periodic selection that regularly purges the genetic diversity in the same ecological niche [14]. As a consequence, ecotypes occupying different niches independently experience selective sweeps, the mutants from one niche not invading the ones from the other niche. Thus, the stability of a cross-feeding polymorphism should only be analyzed in the light of the robustness of each ecotype against selective sweeps by other ecotypes [14]. This mechanism is observed in the LTEE, as well as in our model. In the periodic environment, ecotypes A and B independently experience periodic selection events. In the continuous environment, competitive exclusion implied that only one ecotype evolved in this environment.

Secondly, when ecotypes A and B evolved in the periodic environment were transferred in the continuous environment, they retained their negative frequency-dependent interaction for hundreds of generations, until a selective sweep purged the whole population diversity, and destroyed the cross-feeding interaction. Moreover, ecotypes A and B that evolved for a long time in the periodic environment had a more robust interaction in continuous conditions, because of niche specialization and character displacement on the long-term. In the light of those results, we suggest to distinguish between ecological stability and evolutionary stability. Even if different monophyletic clusters, related by cross-feeding interactions, have frequency-dependent interactions, they are not necessarily robust to competitive exclusion on the long-term. In this sense, ecotypes A and B are no longer ecotypes in the continuous environment. By contrast, in the periodic environment, A and B ecotypes can be considered as proto-species.

Those remarks lead us to hypothesize that the S and L interaction observed in the LTEE, which is still at an early stage, should not be stable in a chemostat on the long-term, even if it could become more and more stable. We also hypothesize that the S/L polymorphism is an ongoing speciation event. On the long run, the S ecotype could even loose the ability to consume glucose.

In a more general view, what we observed is strongly related to known results about temporal niche partitioning in ecology [17]. Bacterial communities commonly undergo adaptive diversification or niche specialization in sympatry, when the environment is seasonal. For example, this mechanism has been observed in marine microbial communities [42], and in lake phytoplankton [43]. In the LTEE [13] and in our model, seasonality of glucose originates from the serial transfer, but the seasonality of acetate is due to cross-feeding and niche construction. Moreover, we demonstrated in our model than negative frequency-dependent cross-feeding is not enough to stabilize the interaction between multiple ecotypes. External factors are necessary, such a regular serial transfer. While the environment is intentionally simplified in those experiments, we can expect much more complex environmental conditions in nature.

Such complex interactions between external factors, emergent cross-feeding interactions and niche construction are therefore of primary importance to understand the evolution of microbial communities in well-mixed environments. Using a computational model of ISEE to decipher those interactions seems to be a rich complementary approach to wet experiments and mathematical modeling.

## Conclusion

Using a multi-scale computational model of ISEE, we studied the evolution and stability of cross-feeding interactions in well-mixed environments, providing a single limiting resource periodically or continuously, as in batch cultures or chemostat devices. Our results led us to consider a stable cross-feeding polymorphism as the stable coexistence of different ecotypes, defined as different monophyletic clusters undergoing independent periodic selection events in their own ecological niche [14]. We observed that, even if cross-feeding polymorphism systematically appears in all the simulations, the evolution of stable ecotypes coexisting via cross-feeding is favored in the periodic environment, similarly to the S/L polymorphism observed in the LTEE [11]. In the continuous environment, competitive exclusion precludes the stabilization of cross-feeding interactions, in apparent contradiction with wet experiments. Indeed, while ecotypes interacting via cross-feeding can temporarily coexist, a mutant always eventually outcompetes them. Then, we suggest to study the evolution of cross-feeding polymorphism by fully integrating the notion of ecotype, and distinguishing between ecological stability and evolutionary stability, the latter including long-term evolutionary dynamics such as periodic selection. Our results contributed to understand temporal niche partitioning, by modeling various mechanisms such as cross-feeding, niche construction and seasonality. At a more general scale, our results may contribute to the study of the evolution of bacterial communities, by deciphering the conditions of sympatric speciation in asexual populations.

## Supporting information

S1 Table Common simulation parameters for the entire experimental protocol. (PDF)

S1 Fig Final phylogenetic trees of each simulation. **(A)** Phylogenetic trees of the 12 repetitions in the periodic environment. **(B)** Phylogenetic trees of the 12 repetitions in the continuous environment. Tree leaves are colored depending on their trophic group: group A in blue, group B in green. Phylogenetic trees are numbered by repetition. For each tree, the scale is represented in simulation time-steps.
(TIF)

S2 Fig Evolution of the MRCA age during simulations, for the three types of environments. For each environment, all the repetitions are represented in different colors. **(A)** Periodic environment. **(B)** Random environment. **(C)** Continuous environment.
(TIF)

S3 Fig Loss of essential metabolites production of ecotype A organisms, in the 10 repetitions of the early population 3 in the continuous environment assays. The 10 repetitions of population 3 are displayed. The 7 essential metabolites (2, 3, 5, 11, 13, 17, and 23) that were produced by ecotype A organisms at the beginning of the assays are represented vertically for each repetition. Background colors indicate a production loss. Essential metabolites that are consumed by ecotype B organisms are colored in red, the other in green. At the top, the evolution of groups A and B proportions is represented, and is colored in green when the A/B interaction persisted, or in red when the interaction failed. In all simulations where A have ceased to produce a metabolite pumped-in by B, B has gone to extinction.
(TIF)

S1 Appendix Sensitivity analysis on six key parameters. Six key parameters have been explored to evaluate the probability of emergence of a stable cross-feeding in the periodic and continuous environments. Each parameter has been modified independently around default parameter values. The emergence of a stable cross-feeding is evaluated in the same way as in Fig 5 (PS score against MRCA age).
(PDF)

S1 Video Variation of the relative fitness of ecotype B during an entire cycle. This video shows the evolution of the ecotype B relative fitness during the first cycle of the short-term competition experiment. Each of the 333 frames corresponds to one time-step, the whole video presenting the entire cycle.
(MPG)
