# Supplementary material for: Beware batch culture: Seasonality and niche construction predicted to favor bacterial adaptive diversification
Source: PLoS Comput Biol. 2017 Mar 30;13(3):e1005459. doi: 10.1371/journal.pcbi.1005459 (PMC5391122; doi:10.1371/journal.pcbi.1005459)
Supplement: S1 Table — (PDF) [file pcbi.1005459.s001.pdf]

S1 Table - Simulation parameters common to the whole experimental protocol.

Parameters for the initialization of genomes	Value	Unit
Initial number of non-coding units (NC)	10	genomic-units
Initial number of promoter units (P)	10	genomic-units
Initial number of enzyme units (E)	10	genomic-units
Range for the random drawing of β in initial genes	$[0, 1]$	ACU.centi-time-step^{-1}
Range for the random drawing of s and p in initial genes	#1 to #20	dimensionless
Range for the random drawing of k_{cat} in initial genes	$[10^{-3}, 10^{-1}]$	centi-time-step^{-1}
Range for the random drawing of k_{cat}/K_M ratio in initial genes	$[10^{-5}, 10^{-4}]$	centi-time-step^{-1}.ACU^{-1}
Parameters of the intracellular dynamics	Value	Unit
Duration of one population time-step	100	centi-time-steps
Protein degradation rate ϕ	0.1	centi-time-step^{-1}
Non essential metabolites toxicity threshold	1.0	ACU
Essential metabolites toxicity threshold	1.0	ACU
Minimum score	10^{-3}	ACU
Parameters of population dynamics	Value	Unit
Total simulation time	500,000	time-steps
Grid width W	32	gridsteps
Grid height H	32	gridsteps
Death probability p_{death}	0.02	organism^{-1}.time-step^{-1}
Metabolite tag of the primary resource m_{exo}	#10	dimensionless
Diffusion parameter D	0.1	gridstep^2.time-step^{-1}
Parameters of point mutations	Value	Unit
Point mutation rate	1e-03	attribute^{-1}replication^{-1}
Substrate tag mutation size	1	dimensionless
Product tag mutation size	1	dimensionless
$log(k_{cat})$ tag mutation size	0.1	dimensionless
$log(k_{cat}/K_M)$ tag mutation size	0.1	dimensionless
β mutation size	0.1	ACU.centi-time-step^{-1}
Probability that a genomic unit changes type	1e-03	genomic-unit^{-1}.replication^{-1}
Parameters of genomic rearrangements	Value	Unit
Duplication rate	1e-03	genomic-unit^{-1}.replication^{-1}
Deletion rate	1e-03	genomic-unit^{-1}.replication^{-1}
Translocation rate	1e-03	genomic-unit^{-1}.replication^{-1}
Inversion rate	1e-03	genomic-unit^{-1}.replication^{-1}
Probability of attribute swap at breakpoint	1e-03	attribute^{-1}.breakpoint^{-1}
Maximum genome size	10000	genomic units

Those parameters are common to all the simulations of the experimental protocol.
