# Supplementary figures and images for: Beware batch culture: Seasonality and niche construction predicted to favor bacterial adaptive diversification
Source: PLoS Comput Biol. 2017 Mar 30;13(3):e1005459. doi: 10.1371/journal.pcbi.1005459 (PMC5391122; doi:10.1371/journal.pcbi.1005459)

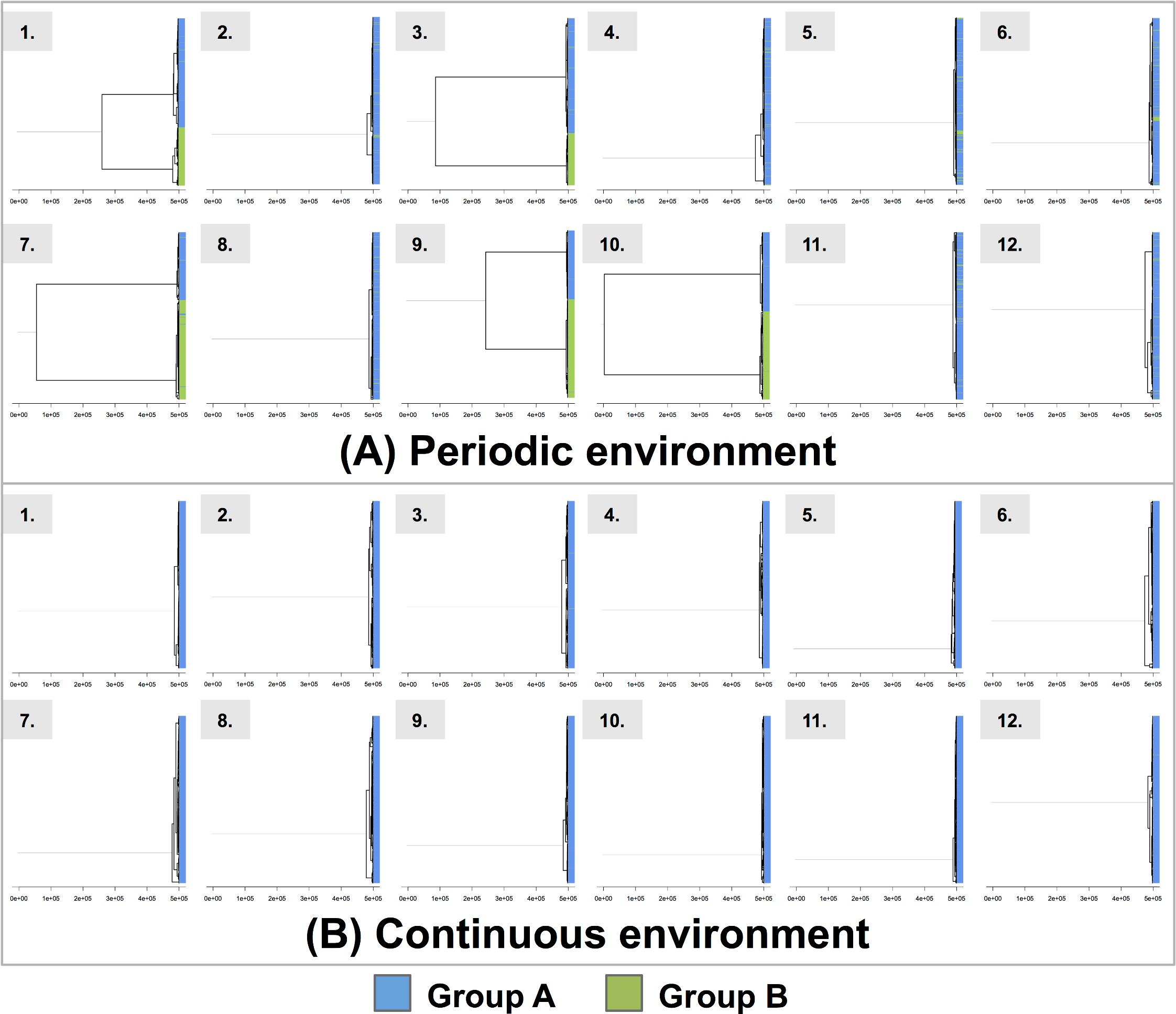

Supplement: S1 Fig — (A) Phylogenetic trees of the 12 repetitions in the periodic environment. (B) Phylogenetic trees of the 12 repetitions in the continuous environment. Tree leaves are colored depending on their trophic group: group A in blue, group B in green. Phylogenetic trees are numbered by repetition. For each tree, the scale is represented in simulation time-steps. (TIF) [file pcbi.1005459.s002.tif]

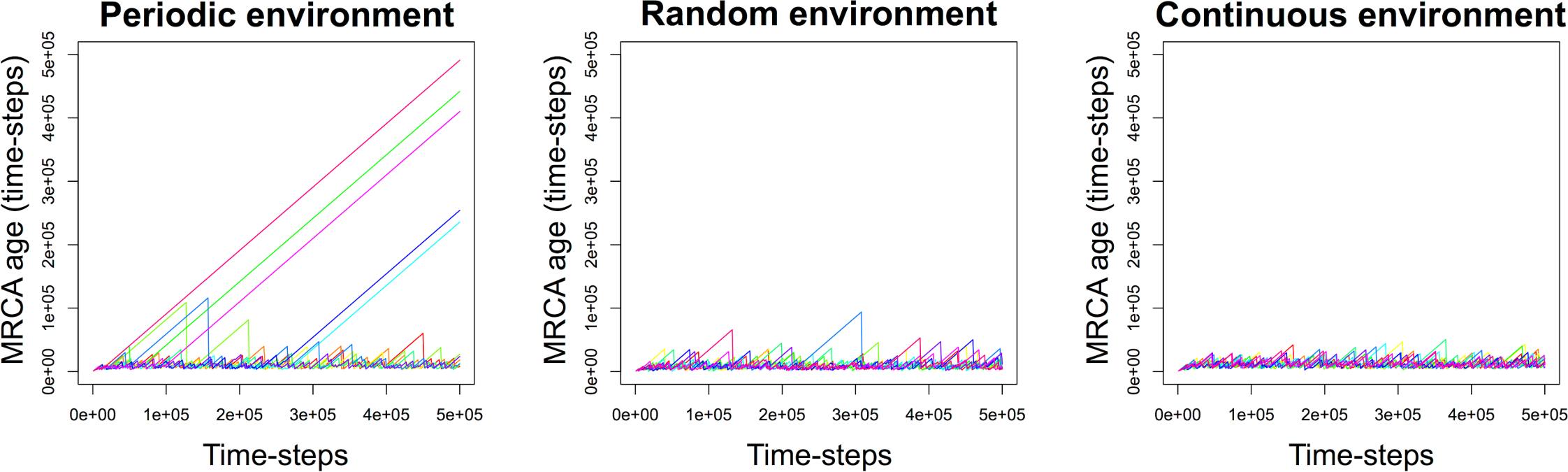

Supplement: S2 Fig — For each environment, all the repetitions are represented in different colors. (A) Periodic environment. (B) Random environment. (C) Continuous environment. (TIF) [file pcbi.1005459.s003.tif]

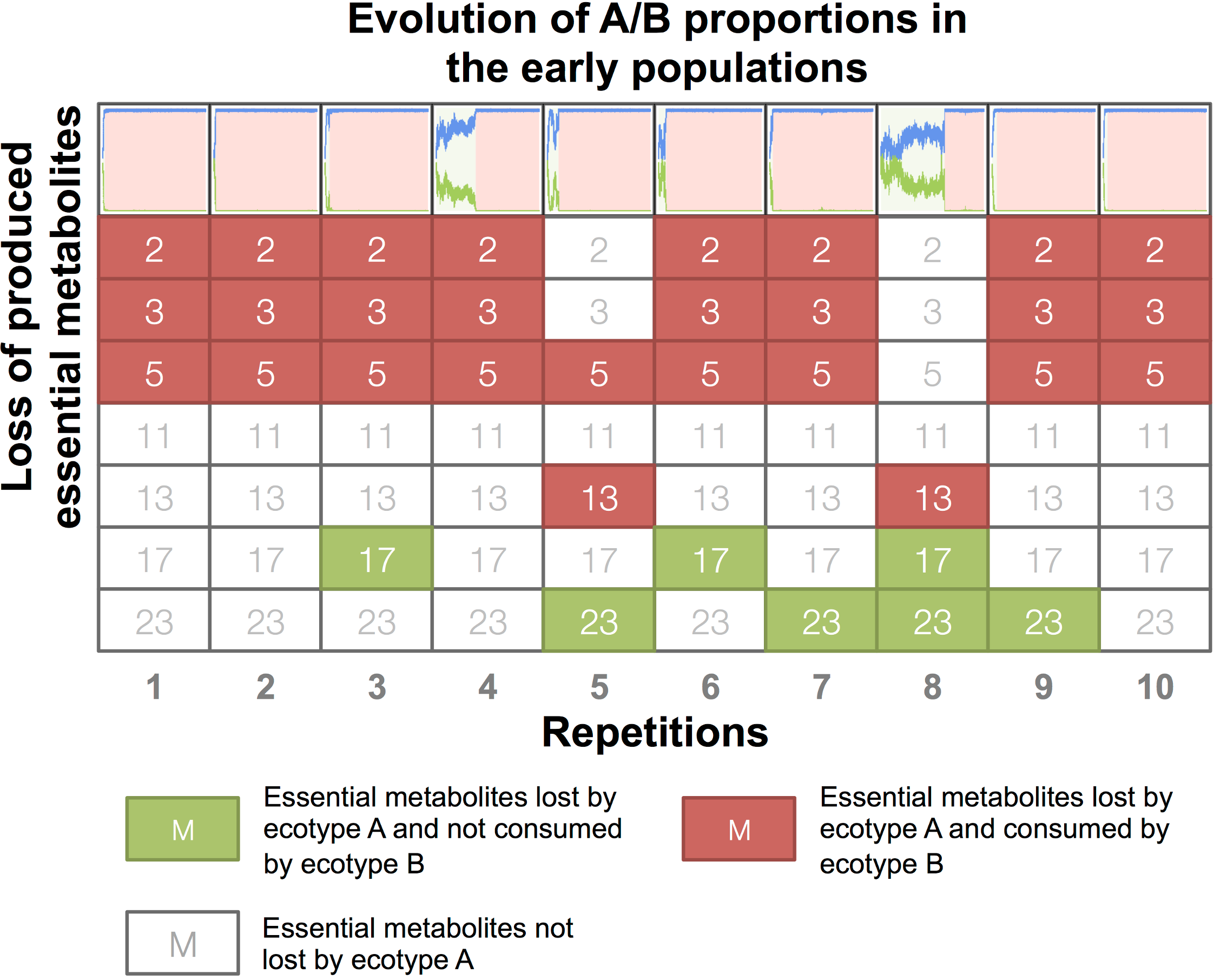

Supplement: S3 Fig — The 10 repetitions of population 3 are displayed. The 7 essential metabolites (2, 3, 5, 11, 13, 17, and 23) that were produced by ecotype A organisms at the beginning of the assays are represented vertically for each repetition. Background colors indicate a production loss. Essential metabolites that are consumed by ecotype B organisms are colored in red, the other in green. At the top, the evolution of groups A and B proportions is represented, and is colored in green when the A/B interaction persisted, or in red when the interaction failed. In all simulations where A have ceased to produce a metabolite pumped-in by B, B has gone to extinction. (TIF) [file pcbi.1005459.s004.tif]
