# Supplementary material for: Beware batch culture: Seasonality and niche construction predicted to favor bacterial adaptive diversification
Source: PLoS Comput Biol. 2017 Mar 30;13(3):e1005459. doi: 10.1371/journal.pcbi.1005459 (PMC5391122; doi:10.1371/journal.pcbi.1005459)
Supplement: S1 Appendix — Six key parameters have been explored to evaluate the probability of emergence of a stable cross-feeding in the periodic and continuous environments. Each parameter has been modified independently around default parameter values. The emergence of a stable cross-feeding is evaluated in the same way as in Fig 5 (PS score against MRCA age). (PDF) [file pcbi.1005459.s005.pdf]

Beware Batch Culture: Seasonality and Niche Construction Predicted to Favor Bacterial Adaptive Diversification
S1 Appendix - Sensitivity analysis on model key parameters

Charles Rocabert[1], Carole Knibbe[1], Jessika Consuegra[2,3], Dominique Schneider[2,3], Guillaume Beslon[1*]

1 Univ. de Lyon, CNRS, INRIA, INSA-Lyon, UCB Lyon 1, LIRIS UMR5205, F-69622 Lyon, France.

2 Univ. Grenoble Alpes, Laboratoire Techniques de l'Ingénierie Médicale et de la Complexité - Informatique, Mathématiques et Applications, Grenoble (TIMC-IMAG), F-38000 Grenoble, France.

3 Centre National de la Recherche Scientifique (CNRS), TIMC-IMAG, F-38000 Grenoble, France.

* guillaume.beslon@inria.fr

To evaluate the robustness of our results to the variation of main simulation parameters, we performed a sensitivity analysis. We explored six key parameters, selected for their importance and their influence on evolutionary dynamics:

(1) The death probability p_{death}, that controls the probability to die at each simulation time-step (the same for every individuals, see "Population and environment" section). We explored p_{death} around the default value ($p_{death} = 0.02$), *i.e.*, $p_{death} = 0.005$ and $p_{death} = 0.1$. Because this parameter is highly sensitive, we also explored intermediate values, *i.e.*, $p_{death} = 0.01$ and $p_{death} = 0.05$

(2) The diffusion rate (see "Population and environment" section). We explored the diffusion rate around the default value (diffusion parameter $D = 0.1$ gridstep^2.time-step^{-1}), *i.e.*, $D = 0.02$ gridstep^2.time-step^{-1} and the special condition of a perfectly well-mixed environment.

(3) The mutation rates (see "Genome structure" section). We explored the mutation rates around the default value (10^{-3}), *i.e.*, 2.10^{-4} and 5.10^{-3}.

(4) The toxicity thresholds, that impose a lethal upper threshold to internal cell's metabolic concentrations. We explored the mutation rates around the default value (1.0), *i.e.*, 0.1 ACU (Arbitrary Concentration Unit) and 5.0 ACU.

(5) The “migration rate" r_{mig}: this parameter controls, at each time-step, the fraction of exchanged pairs among all possible pairs of individuals. As competition is local, this parameter thus controls whether an individual directly compete with its siblings or with more distantly related individuals. By default, the migration rate is $r_{mig} = 0.0$. We then explored this parameter with to higher values: $r_{mig} = 0.5$ (half of the locations are randomized) and $r_{mig} = 1.0$ (every locations are randomized).

(6) The grid size. The default grid size being 32×32, we tested sizes 20×20 and 50×50.

Because of computational loads, we varied each parameter separately around our default parameters set. We computed 10 repetitions for each set of values (with a total of 140 simulations of 500,000 time steps and 1.5 months of computation).

To assess whether a A/B-like stable polymorphism evolved in a given run, we analyzed the phylogenetic tree of the final population and computed both the PS score (see the paragraph on cross-feeding interactions in the manuscript) and the time to the most recent common ancestor (MRCA age). As in Fig 5, we considered that a A/B stable polymorphism had evolved if **(i)** the MRCA age was higher than 200,000 time-steps, which indicates the existence of long-diverged clades, and **(ii)** the PS score was higher than 0.9, which indicates that clades match well with ecotypes (ecotypes are monophyletic). The result of the sensitivity is presented in Fig 1.

• First, no stable polymorphism evolved in the continuous environment, whatever the parameter values, while it regularly evolved in the periodic environment, thereby supporting our main conclusion.

• Second, in the periodic environment, some parameters are more sensitive than others in the periodic environment.

(i) For example, a lower or higher death probability (Figs 1A.1 and 1B.1) inhibits the emergence of stable polymorphism. Interestingly, we calibrated the death probability (0.02 per organism per time-step) and the duration of a cycle in the periodic environment (333 times-steps) to obtain in theory 6.67 generations per cycle - since $333 * 0.02 = 6.67$ - like in the LTEE. A lower death probability exposes individuals to several seasons, facilitating the evolution of generalists. On the opposite, a higher death rate forbids the survival of B individuals, that would have too short a lifespan to survive the famine that (for them) necessarily follows the environment refresh.

(ii) Lower or higher toxicity thresholds (Figs 1A.4 and 1B.4) strongly influence the structuring of the metabolic network, and then the ability for the A/B interaction to be stable (see part "Stability of the A/B cross-feeding interactions in the continuous environment" of the manuscript for details).

(iii) The variation of the mutation rates (Figs 1A.3 and 1B.3) also influences the outcome of the simulations. Stable polymorphism is observed at higher mutation rate but not at a lower one which may be due to a too slow evolution rate compared with the duration of the experiment (structured trees are indeed observed at a low mutation rate but the MRCA ages remain low).

• Third, and most importantly, the exploration of the diffusion rate (Figs 1A.2 and 1B.2) and of the migration rate (Figs 1A.5 and 1B.5) reinforces our conclusions. Indeed, previous theoretical studies highlighted the fact that spatial structure could inhibit the emergence of stable polymorphism, while well-mixed conditions could enhance it (Hauert & Doebeli, 2004 ; Gerlee & Lundh, 2012). In the case of the periodic environment, there is a clear correlation between the rate of diffusion and the number of simulations exhibiting stable polymorphism ($D = 0.02 \rightarrow 0\%$, $D = 0.1 \rightarrow 42\%$, well-mixed$\rightarrow$ 80%). Conclusions are the same for the migration rate ($r_{mig} = 0.0 \rightarrow 42\%$, $r_{mig} = 0.5 \rightarrow 50\%$, $r_{mig} = 1.0 \rightarrow 100\%$). This result is in agreement with previous studies (Hauert & Doebeli, 2004 ; Gerlee & Lundh, 2012). However, there is no stable polymorphism in the continuous environment in any cases, thus reinforcing our conclusion that stable polymorphism cannot evolve on the long-term in chemostat-like environments because of competitive exclusion.

• We also explored the grid size, by decreasing the grid size to 20×20, and increasing it to 50×50 (Figs 1A.6 and 1B.6). A small grid size (20×20) seems to inhibit the emergence of a stable polymorphism.

Indeed, the population size is probably too small to sustain the polymorphism. In the large grid, we observed a slightly less stable polymorphism (30%) than in the default grid (42%), but the difference is not significant.

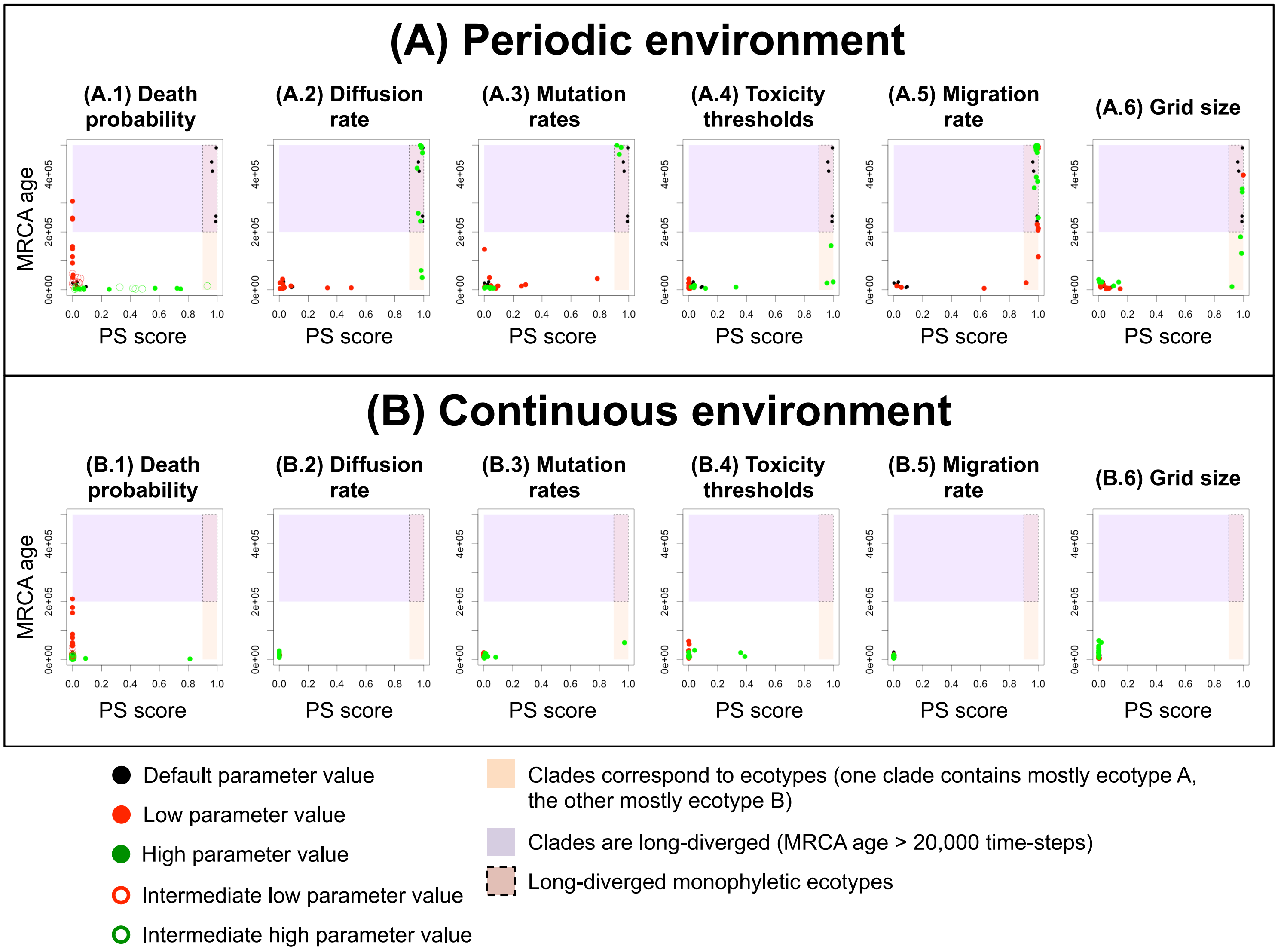

Figure 1 - **Sensitivity analysis on six key parameters of Evo²Sim.** Six parameters are explored: **(i)** the death probability p_{death}, **(ii)** the di usion rate, **(iii)** the mutation rates, **(iv)** the toxicity thresholds, **(v)** the migration rate and **(vi)** the grid size. For each type of environment, the PS score is plotted against the MRCA age for the nal trees (at 500,000 time-steps): **(i)** default parameter value simulations are plotted in black, **(ii)** low parameter value simulations are plotted in red, **(iii)** high parameter value simulations are plotted in green. **(iv)** For p_{death}, intermediates low and high values are also explored (resp. red and green circles). On each plot, three areas are identi ed: purple area indicates long-diverged clades (MRCA age higher than 200,000 time-steps), orange area indicates that clades correspond to ecotypes (PS score $>$ 0.9). The intersection of the two previous areas (inside dashed borders) indicates long-diverged monophyletic ecotypes. **(A)** Periodic environment. **(B)** Continuous environment.
